# The G4 resolvase RHAU modulates mRNA translation and stability to sustain postnatal heart function and regeneration

**DOI:** 10.1074/jbc.RA120.014948

**Published:** 2020-11-23

**Authors:** Mingyang Jiang, Han Hu, Ke Zhao, Ruomin Di, Xinyi Huang, Yingchao Shi, Yunyun Yue, Junwei Nie, Shan Yu, Wengong Wang, Zhongzhou Yang

**Affiliations:** 1 State Key Laboratory of Pharmaceutical Biotechnology, Department of Cardiology, Nanjing Drum Tower Hospital, The Affiliated Hospital of Nanjing University Medical School and MOE Key Laboratory of Model Animal for Disease Study, Model Animal Research Center, Nanjing University, Nanjing, China; 2 Department of Biochemistry and Molecular Biology, School of Basic Medical Sciences, Peking University Health Science Center, Beijing, China; 3 Department of Cardiology, The Fifth People’s Hospital of Shanghai, Fudan University, Shanghai, China

**Keywords:** RHAU, RNA-binding protein, heart, mRNA, HEXIM1, YAP1, regeneration, AREs, adenylate- and uridylate-rich elements, CDS, coding sequence, DCM, dilated cardiomyopathy, EGFP, enhanced GFP, FC, fold change, G4, G-quadruplex, GO, gene ontology, HEK293, human embryonic kidney 293 cells, HEXIM1, hexamethylene bis-acetamide inducible 1, KEGG, Kyoto Encyclopedia of Genes and Genomes, MI, myocardial infarction, Myh7, myosin, heavy polypeptide 7, Nppa, natriuretic peptide type A, Nppb, natriuretic peptide type B, RBPs, RNA-binding proteins, RHAU, RNA helicase associated with adenylate- and uridylate-rich element, Rhau-cKO, Rhau conditional knockout, RIP, ribonucleoprotein immunoprecipitation, YAP1, yes1-associated transcriptional regulator

## Abstract

Post-transcriptional regulation of mRNA translation and stability is primarily achieved by RNA-binding proteins, which are of increasing importance for heart function. Furthermore, G-quadruplex (G4) and G4 resolvase activity are involved in a variety of biological processes. However, the role of G4 resolvase activity in heart function remains unknown. The present study aims to investigate the role of RNA helicase associated with adenylate- and uridylate-rich element (RHAU), an RNA-binding protein with G4 resolvase activity in postnatal heart function through deletion of *Rhau* in the cardiomyocytes of postnatal mice. RHAU-deficient mice displayed progressive pathological remodeling leading to heart failure and mortality and impaired neonatal heart regeneration. RHAU ablation reduced the protein levels but enhanced mRNA levels of *Yap1* and *Hexim1* that are important regulators for heart development and postnatal heart function. Furthermore, RHAU was found to associate with both the 5' and 3' UTRs of these genes to destabilize mRNA and enhance translation. Thus, we have demonstrated the important functions of RHAU in the dual regulation of mRNA translation and stability, which is vital for heart physiology.

In eukaryotic cells, RNA is steadily transcribed, translated, and degraded to serve various crucial cellular functions; this process is rigorously regulated (1, 2). Numerous prior studies have been performed to comprehensively investigate the regulatory mechanisms pertaining to the particular events of RNA biology/metabolism from two perspectives: the transcriptional and the post-transcriptional levels (3, 4). At the transcriptional level, RNA transcription (gene expression) uses protein complexes to control DNA methylation and histone modification (5), whereas the post-transcriptional regulation engages RNA-binding proteins (RBPs) to govern mRNA translation and stability (degradation/decay) (6).

RBPs are highly conserved in eukaryotes, and more than 1500 RBPs have been identified in humans (7, 8). Among these RBPs, approximately 700 RBPs bind to mRNA and are therefore designated mRBPs (9). mRBPs play a critical role in post-transcriptional regulation of mRNA translation, stability, and subcellular localization (10). For instance, most mRNAs are translated by a scanning mechanism wherein the m^7^G cap at the 5'-UTR is bound with the cap-binding complex eIF4F to activate mRNA (11, 12). The eIF4F complex is composed of the cap-binding protein eIF4E, eIF4G, and the RNA helicase eIF4A, all of which are mRBPs (12). On the other hand, the adenylate- and uridylate (AU)-rich elements (AREs) at the 3'-UTR of mRNAs are associated with specific mRBPs for stability control (13).

RNA helicase associated with AREs (RHAU) (also termed DHX36) is an mRBP and was originally identified by its high binding affinity for the AREs in certain RNA molecules (14, 15). Later on, it was found that RHAU possesses resolvase activity to unwind the G-quadruplex (G4) structures in DNA and RNA; therefore, it was alternatively called G4 resolvase 1 (16, 17, 18). RHAU is essential for embryonic development in that the global knockout of *Rhau* causes early embryonic lethality at around embryonic day 7.5 (19). Genetic study of *Rhau*-deletion mice also revealed a critical role of RHAU in hematopoiesis, particularly erythropoiesis, and in spermatogonia differentiation (19, 20).

In a previous study, we revealed the crucial function of RHAU in embryonic heart development in mice (21). Deletion of *Rhau* in either the cardiac mesoderm or the cardiomyocyte progenitors results in severe heart defects and embryonic lethality by midgestation. Mechanistically, RHAU was identified to associate with both the 5'-UTR and the 3'-UTR of *Nkx2-5* (NK2 homeobox 5) mRNA to regulate mRNA translation and stability. The association of RHAU with the G4 structure in the 5'-UTR of *Nkx2-5* mRNA facilitates translation, whereas binding to the ARE in the 3'-UTR of the same mRNA promotes degradation (21). Thus, we deciphered a new regulatory mechanism that controls the translation and stability of the same mRNA molecule. However, whether this type of dual regulation of mRNA translation and stability is specific to *Nkx2-5* mRNA or a general governing principle will require further investigation.

Mouse genetics studies have demonstrated that both hexamethylene bis-acetamide inducible 1 (HEXIM1) and yes1-associated transcriptional regulator (YAP1) are essential for embryonic heart development and postnatal heart function. *Hexim1*-null mice are embryonic lethal with aberrant heart development (22). *Hexim1* is also expressed in postnatal organs and/or tissues with the highest amount in the heart, brain, and skeletal muscles (22). An insertional mutation in the *Hexim1* gene resulting in disrupted C-terminal region in mice causes thin ventricular wall, and inducible postnatal expression of *Hexim1* in the cardiomyocytes gives rise to physiological cardiac hypertrophy (23, 24). These studies suggest of an important role of HEXIM1 in promoting heart growth. Cardiac-specific deletion of *Yap1* during embryonic stage using *Tnnt2-Cre* and *Nkx2.5-Cre* lines has revealed severe heart defects (25, 26). Similarly, postnatal deletion of *Yap1* in the heart causes dilated cardiomyopathy (DCM) and premature death (27). Meanwhile, it was found that YAP1 stimulates heart growth mainly through modulating cardiomyocyte proliferation (25).

In the present study, we set out to investigate the role of RHAU in postnatal heart physiology and diseases and to explore its dual regulation on mRNA translation and stability. For this purpose, we deleted *Rhau* specifically in the cardiomyocyte of postnatal mouse heart. RHAU deficiency causes progressive pathological heart remodeling that leads to heart failure and mortality within 6 months and impaired heart regeneration in neonatal mice. We found that the dual regulation of mRNA translation and stability is disrupted for a panel of genes, including *Yap1*, *Hexim1*, and *Nkx2-5*, which play crucial roles in heart growth and remodeling. Thus, we have demonstrated the crucial functions of RHAU in regulating the translation and stability of the same mRNA molecules, which is important for postnatal heart physiology.

## Results

### *Rhau* deletion in postnatal cardiomyocytes causes DCM and heart failure

**Figure 1:**
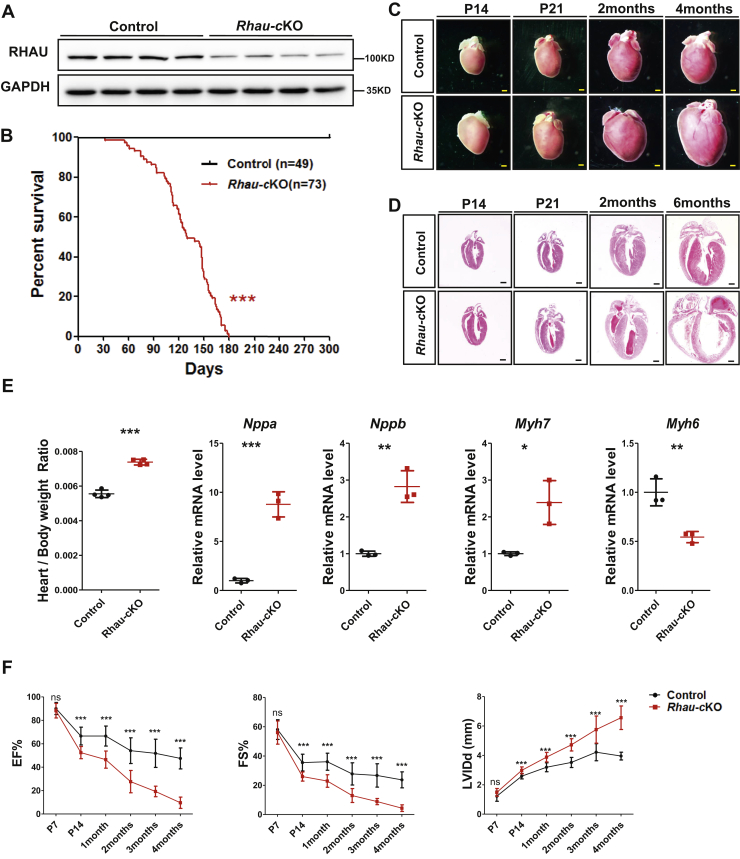
***Rhau* deletion induced dilated cardiomyopathy and heart failure.***A*, western blotting analysis confirmed effective *Rhau* deletion in *Rhau*-cKO (*α-MHC-Cre*;*Rhau*^*F/F*^) mouse hearts at P7. Littermates (*Rhau*^*F/F*^) were used as controls. GAPDH was used as a loading control. *B*, survival curves. N = 49 for control (*Rhau*^*F/F*^) mice and n = 73 for *Rhau*-cKO (*α-MHC-Cre;Rhau*^*F/F*^) mice. *C*, heart morphology of control (*Rhau*^*F/F*^) mice and *Rhau*-cKO (*α-MHC-Cre;Rhau*^*F/F*^) mice at P14, P21, 2 months, and 4 months. The scale bar represents 0.5 mm. *D*, histological analysis for control (*Rhau*^*F/F*^) mice and *Rhau*-cKO (*α-MHC-Cre;Rhau*^*F/F*^) mice at P14, P21, 2 months, and 6 months. The scale bar represents 0.5 mm. *E*, heart/body weight ratio analysis (n = 4) and quantitative RT-PCR (n = 3) analysis of cardiac hypertrophic markers in control (*Rhau*^*F/F*^) mice and *Rhau*-cKO (*α-MHC-Cre;Rhau*^*F/F*^) mice at P21. *F*, echocardiographic analysis of control (*Rhau*^*F/F*^) mice and *Rhau*-cKO (*α-MHC-Cre;Rhau*^*F/F*^) mice at various ages. EF, FS, and LVIDd were calculated according to the guidelines accompanying the Vevo 770 UBM system. Numbers of mice are as follows. *Control group*: P7 (n = 10), P14 (n = 14), 1 month (n = 9), 2 months (n = 11), 3 months (n = 9), and 4 months (n = 9); *Rhau-cKO mice*: P7 (n = 13), P14 (n = 11), 1 month (n = 9), 2 months (n = 11), 3 months (n = 9), and 4 months (n = 9). EF, ejection fraction; FS, fractional shortening; LVIDd, left ventricular internal diameter at end diastole; ns, not significant; RHAU, RNA helicase associated with adenylate- and uridylate-rich element.

To investigate the role of RHAU in postnatal heart function, we deleted *Rhau* in the cardiomyocytes of postnatal mice. The *α-MHC-Cre* line is commonly used to inactivate gene specifically in postnatal cardiomyocytes; therefore, we crossed *Rhau* floxed mice (*Rhau*^*F/F*^) with the *α-MHC-Cre* line to generate *Rhau* conditional knockout (*α-MHC-Cre*;*Rhau*^*F/F*^) mice (hereafter referred as *Rhau*-cKO). Western blotting analysis confirmed sufficient *Rhau* deletion in the hearts of *Rhau*-cKO mice at P7 (7 days after birth) (Fig. 1*A*). To further confirm the efficiency of *Rhau* ablation specifically in cardiomyocytes, we isolated cardiomyocytes from *Rhau-cKO* and control mice at 2 months for Western blotting analysis. The results demonstrated highly effective RHAU knockout in *Rhau-cKO* mice heart (Fig. S1).

*Rhau*-cKO mice were viable and born at the expected Mendelian ratios (data not shown). However, all *Rhau*-cKO mice were lost within 6 months (Fig. 1*B*). Anatomical and histological analysis of the hearts indicated that ablation of *Rhau* induced progressive DCM (Fig. 1, *C*–*D*). The heart/body weight ratio was significantly increased in *Rhau*-cKO mice, and molecular profiling for the markers of pathological heart remodeling revealed profoundly enhanced expression levels of *Nppa* (natriuretic peptide type A), *Nppb* (natriuretic peptide type B), and *Myh7* (myosin, heavy polypeptide 7, cardiac muscle, beta), but a substantially reduced expression level of *Myh6* (myosin, heavy polypeptide 6, cardiac muscle, alpha), indicating apparent pathological heart remodeling (Fig. 1*E*) (28). In agreement with those results, echocardiography analysis displayed progressively reduced heart contractility but dramatically increased left ventricular inner diameter in *Rhau*-cKO mice (Fig. 1*F*).

Furthermore, we performed wheat germ agglutinin staining to evaluate the effects of *Rhau* ablation on the size of cardiomyocytes. The results showed that deletion of *Rhau* caused a 1.68-fold enlargement of cardiomyocytes by 2 months of age compared with those of controls (Fig. S2*A*). TUNEL staining revealed significantly enhanced cell death in the cKO hearts at 3 months (Fig. S2*B*).

In addition, we crossed *Rhau*^*F/F*^ mice with the *CAAG-ERT2-Cre* (*ERT2-Cre*) line to generate *ERT2-Cre;Rhau*^*F/F*^ mice for chemically inducible deletion of *Rhau* in the adults. On five successive doses of tamoxifen administration to control and *ERT2-cre;Rhau*^*F/F*^ mice at 2 months of age, *Rhau* was effectively deleted in the hearts of *ERT2-cre;Rhau*^*F/F*^ mice as demonstrated by Western blotting analysis (Fig. S3*A*). Histological analysis revealed mild cardiac dilation in the mice 1 month after tamoxifen administration (Fig. S3*B*). However, 2 months after tamoxifen administration, the hearts of *ERT2-cre;Rhau*^*F/F*^ mice were severely dilated compared with those of littermate controls (*Rhau*^*F/F*^), and their contractile function was dramatically impaired (Fig. S3, *B*–*D*). Collectively, these results reveal that RHAU is required for postnatal heart growth and maintenance of cardiac function.

### *Rhau* deletion results in disrupted mRNA and protein levels of YAP1 and HEXIM1

**Figure 2:**
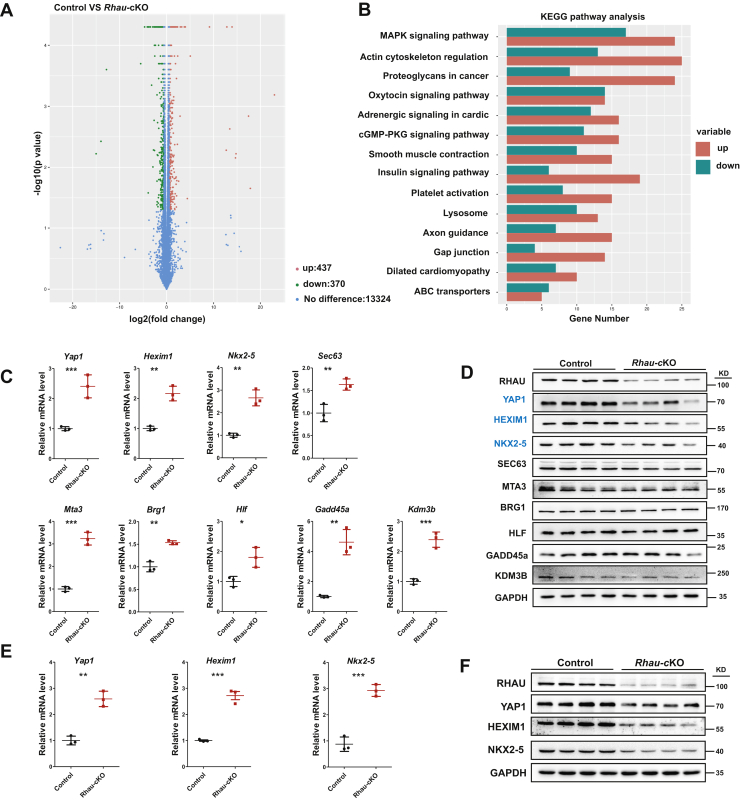
**Rhau deletion results in disrupted mRNA and protein levels of *YAP1* and *HEXIM1*.***A*, volcano plot of RNA-Seq analysis. RNA was isolated from the hearts of control (*Rhau*^*F/F*^) and *Rhau*-cKO (*α-MHC-Cre;Rhau*^*F/F*^) mice at P10. Differentially expressed genes were filtered with fold change ≥1.5 and *p* < 0.05. *B*, KEGG pathway analysis of differentially expressed genes identified from RNA-Seq. *C*, quantitative RT-PCR analysis of candidate genes in the hearts of control (*Rhau*^*F/F*^) mice and *Rhau*-cKO (*α-MHC-Cre;Rhau*^*F/F*^) mice at P10. N = 3 for each group. *D*, western blotting analysis of candidate genes in the hearts of control mice and *Rhau*-cKO mice at P10. GAPDH was used as loading controls. *E*, quantitative RT-PCR analysis to detect *Yap1*, *Hexim1*, and *Nkx2-5* mRNA levels. RNA was isolated from the hearts of control (*Rhau*^*F/F*^) and *Rhau*-cKO (*α-MHC-Cre;Rhau*^*F/F*^) mice at P21. N = 3 for each group. *F*, western blotting analysis examined the protein levels of YAP1, HEXIM1, and NKX2-5 in the hearts of control (*Rhau*^*F/F*^) mice and *Rhau*-cKO (*α-MHC-Cre;Rhau*^*F/F*^) mice at P21. GAPDH was used as loading controls. KEGG, Kyoto Encyclopedia of Genes and Genomes; MAPK, mitogen-activated protein kinase; PKG, protein kinase G.

RHAU is localized in the cytosol and nucleus of cardiomyocytes (Fig. S4). Our previous study indicates that RHAU modulates the stability of certain mRNA molecules, and *Rhau* deletion causes substantially increased mRNA levels. Therefore, we performed RNA-Seq to compare gene expression profiles between control and *Rhau* cKO mice. RNA was isolated from the hearts of mice at P10 to identify the early responsive genes on *Rhau* deletion while avoiding secondary effects. The volcano plot depicted a total of 807 differentially expressed genes including 437 upregulated genes and 370 downregulated genes (Fig. 2*A*). Kyoto Encyclopedia of Genes and Genomes (KEGG) pathway analysis was conducted and identified the DCM pathway as one of the significantly affected pathways, which is in agreement with the DCM phenotype of *Rhau* deletion mice (Fig. 2*B* and Table S1). Gene ontology (GO) enrichment analysis was performed for the categories of cellular component, molecular function, and biological process to uncover the relationship among differentially expressed genes. Notably, most genes in all the clusters were upregulated, which is consistent with RHAU being an mRNA destabilizing factor as we previously reported (Fig. S5, *A*–*C*) (21).

In addition, mRNA was isolated from the hearts of mice (*ERT2-Cre;Rhau*^*F/F*^ and *Rhau*^*F/F*^ mice) at 2 months after tamoxifen administration for microarray analysis (Fig. S6, *A*–*B*). Heatmap analysis illustrated a comprehensive comparison of differentially expressed genes between the two groups (Fig. S6*A*). Notably, KEGG pathway analysis indicated that the DCM pathway was affected most (Fig. S6*B*).

Next, we made comparisons among the three data sets of RNA-Seq, microarray, and mRNA G4s (29). The mRNA G4s data set was used here as we and others have found that RHAU functions as an RNA helicase to unwind mRNA G4s. These comparisons identified 21 genes shared by all three data sets (Fig. S6*C*), and the shared genes were considered primary candidates for further investigation. Notably, the mRNA levels of all 21 candidate genes were significantly upregulated, according to the fragments per kilobase of exon per million fragments mapped values from RNA-Seq analysis (Table S2). The alteration of mRNA levels was further confirmed by quantitative RT-PCR (qRT-PCR) for most of these genes using heart samples from control and *Rhau*-cKO mice (P10). Among them, nine genes were possibly involved in regulating heart function by literature search, and their mRNA levels were markedly increased in *Rhau*-cKO hearts (Fig. 2*C*).

Next, we performed Western blotting analysis to examine the protein levels of these nine genes in control and *Rhau*-cKO mice (P10) and revealed distinct reduction of YAP1, HEXIM1, and NKX2-5 (Figs. 2*D* and S11), three proteins that have been reported to play critical roles in heart development, growth, and remodeling (22, 24, 26, 30, 31, 32).

Similar but more apparent changes of YAP1, HEXIM1, and NKX2-5 at both the mRNA and protein levels were observed in control and *Rhau*-cKO mice at P21 compared with those at P10 (Fig. 2, *E*–*F*). These changes were also confirmed in the *ERT2-Cre;Rhau*^*F/F*^ adult mice. Five days after tamoxifen injection, hearts were collected to evaluate the mRNA and protein levels for all three candidate genes. Similar patterns were observed for all the candidates, which revealed that these changes were a primary response on *Rhau* deletion (Fig. S7, *A*–*B*).

Collectively, we have identified *Yap1*, *Hexim1*, and *Nkx2-5* as potential RHAU regulatory targets in postnatal hearts.

### RHAU regulates the stability of *Yap1* and *Hexim1* mRNAs

Our previous study has thoroughly explored the mechanisms of RHAU regulation on *Nkx2-5* mRNA stability and translation (21). Therefore, we tested whether RHAU can directly interact with *Yap1* and *Hexim1* mRNAs to modulate their translation and stability in the postnatal heart.

**Figure 3:**
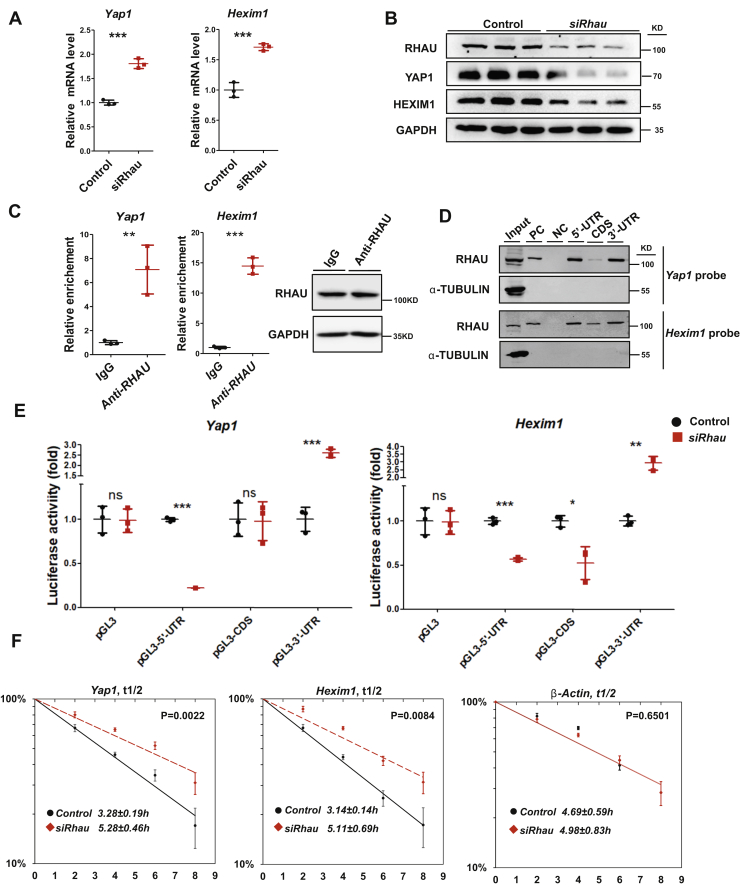
**RHAU regulates the stability of *Yap1* and *Hexim1* mRNAs.***A*, quantitative RT-PCR analysis to measure the mRNA levels of *Yap1* and *Hexim1* in control and *Rhau* knockdown H9C2 cells. *Rhau* was knocked down by transfection of siRNA targeting *Rhau* mRNAs, and control cells were transfected with a negative control siRNA. Seventy-two hours after transfection, cells were harvested. A half of the cells were used for RNA isolation, and the other half of the cells were used for Western blotting analysis. N = 3 for each group. *B*, qestern blotting analysis to detect the protein levels of *RHAU*, *YAP1*, and *HEXIM1* in control and *Rhau* knockdown H9C2 cells. Protein was extracted from the cells described in (*A*). GAPDH was used as loading controls. *C*, ribonucleoprotein immunoprecipitation assays examined the endogenous association of RHAU with *Yap1* and *Hexim1* mRNA in H9C2 cells. The relative enriched levels of *Yap1* and *Hexim1* mRNA were determined by quantitative RT-PCR. The *Gapdh* transcripts were used as internal controls, and IgG antibody was used as negative controls. Western blotting analysis showed the enrichment of RHAU protein levels in the samples. N = 3 for each group. *D*, RNA pull-down assays were performed to assess the association of RHAU with biotinylated 5'-UTRs, CDSs, and 3'-UTRs of *Yap1* and *Hexim1* mRNA. The biotinylated 3'-UTR of *Nkx2-5* mRNA was used as a positive control and biotinylated p27-CDS as a negative control. *E*, luciferase reporter assays explored the effects of RHAU binding to specific fragments of the *Yap1* and *Hexim1* mRNAs on *Rhau* knockdown in H9C2 cells. *Rhau* was knocked down by transfection of siRNA targeting *Rhau* mRNAs, and control cells were transfected with a negative control siRNA. For each column, n = 3. *F*, half-lives of the endogenous mRNAs of *Yap1* and *Hexim1* were examined in *Rhau* knockdown H9C2 cells and control (transfected with a negative control siRNA) cells. The β-actin mRNA was used as an internal control. For each time point, n = 3. IgG, immunoglobulin G; RHAU, RNA helicase associated with adenylate- and uridylate-rich element.

For this purpose, H9C2 cells (a line of cardiomyocyte-like cells) with silenced *Rhau* were subjected to qRT-PCR and Western blotting analysis to assess the protein and mRNA levels of *Yap1* and *Hexim1*, respectively. As shown, knockdown of *Rhau* increased the mRNA levels but reduced protein levels of *Yap1* and *Hexim1* (Fig. 3, *A*–*B*), a pattern of alteration similar to that was observed in the hearts of *Rhau*-deficient mice.

Next, we performed ribonucleoprotein immunoprecipitation (RIP) and RNA pull-down assays to test whether RHAU could directly associate with *Yap1* and *Hexim1* mRNAs. The results indicate that the mRNAs of *Yap1* and *Hexim1* were significantly enriched with anti-RHAU antibody compared with the immunoglobulin G control, indicating robust binding of RHAU to *Yap1* and *Hexim1* mRNAs (Fig. 3*C*). Meanwhile, RNA pull-down assays also demonstrated that RHAU could bind to the 5'-UTRs and 3'-UTRs of *Yap1* and *Hexim1* mRNAs (Fig. 3*D*). In addition, a modest association between RHAU and the coding sequence (CDS) of *Hexim1* mRNA was observed (Fig. 3*D*). Taken together, these results demonstrated direct association of RHAU with *Yap1* and *Hexim1* mRNAs.

Furthermore, through luciferase reporter assays, we studied the effects of the association of RHAU with the fragments of the *Yap1* and *Hexim1* mRNAs. For this purpose, pGL3-Luc-based reporters containing the 5'-UTR, CDS, and 3'-UTR of *Yap1* and *Hexim1* mRNAs were constructed (Fig. S8*A*). On silencing of *Rhau*, the 5'-UTR reporter activity of both *Yap1* and *Hexim1* was decreased dramatically, whereas the 3'-UTR reporter activity was increased profoundly (Fig. 3*E*). Although the pGL3-Luc-CDS activity of *Yap1* was not affected by *Rhau* knockdown, the pGL3-Luc-CDS activity of *Hexim1* was decreased significantly after *Rhau* knockdown (Fig. 3*E*), which could be a result of RHAU binding. It should be noted that addition of 5'-UTRs of *Yap1* and *Hexim1* suppressed, whereas addition of 3'-UTRs of these genes enhanced pGL3 luciferase activity in the presence of control siRNA (possibly because of suppression effects of 5'-UTR and stabilization effects of 3'-UTR). In addition, knockdown of RHAU increased the transcript levels of pGL3-Luc-3'-UTRs of *Yap1* and *Hexim1* but had no effect on those of pGL3-Luc-5'-UTR and pGL3-Luc-CDS for *Yap1* and *Hexim1* (Fig. S8, *B*–*C*). Taken together, RHAU regulates *Yap1* and *Hexim1* expression by association with *Yap1* and *Hexim1* mRNAs. These results suggested that the association of RHAU with the 3'-UTRs of *Yap1* and *Hexim1* may destabilize their mRNAs, whereas association with the 5'-UTRs of *Yap1* and *Hexim1* mRNAs may enhance mRNA translation.

To confirm that RHAU influences the turnover of *Yap1* and *Hexim1* mRNAs, RNA isolated from H9C2 cells at indicated times was subjected to qRT-PCR analysis to assess the half-lives of mRNAs (Fig. 3*F*). As shown, knockdown of RHAU extended the half-lives of *Yap1* and *Hexim1* mRNAs but not that of β-actin mRNA (Fig. 3*F*). Therefore, RHAU destabilized the mRNAs of *Yap1* and *Hexim1*. Taken together, these results indicate that RHAU regulates the stability of *Yap1* and *Hexim1* mRNAs through association with their 3'-UTRs.

### RHAU promotes the translation of *Yap1* and *Hexim1* mRNAs

Because knockdown of *Rhau* reduced the reporter activity of pGL3-Luc-5'-UTR without influencing their transcripts levels, the association of RHAU with the 5'-UTRs of *Yap1* and *Hexim1* mRNAs may influence the mRNA translation.

**Figure 4:**
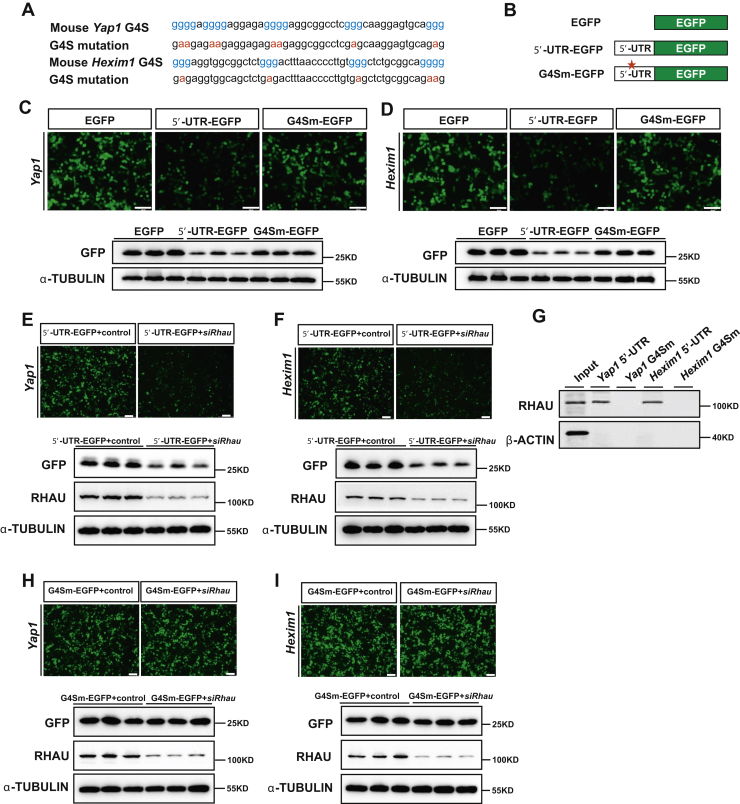
**RHAU promoted the translation of *Yap1* and *Hexim1* mRNAs.***A*, potential G4-forming sequences (G4S) and their corresponding mutations in the 5'-UTRs of *Yap1* and *Hexim1* mRNA are depicted. *B*, schematic representation of the chimeric EGFP vector structure. EGFP represents pEGFP-N1 blank vector; 5'-UTR-EGFP represents chimeric pEGFP-N1 vector flanked with 5'-UTR of *Yap1* or *Hexim1* mRNA, and G4Sm-EGFP represents chimeric pEGFP-N1 vector flanked with G4S-mutated 5'-UTR of *Yap1* or *Hexim1* mRNA. *C* and *D*, representative images show the intensity of chimeric GFP reporters for *Yap1* and *Hexim1* followed by Western blotting analysis to confirm the GFP protein levels. α-Tubulin was used as a loading control. The scale bar represents 200 μm. *E* and *F*, representative images show that *Rhau* knockdown reduced the activity of chimeric GFP reporters for *Yap1* and *Hexim1* followed by Western blotting analysis. α-Tubulin was used as a loading control. The scale bar represents 200 μm. *G*, RNA pull-down assays were performed to assess the association of RHAU with biotinylated 5'-UTRs and G4S-mutated 5'-UTRs. *H* and *I*, representative images show the activity of G4Sm-EGFP reporters for *Yap1* and *Hexim1* in response to *Rhau* knockdown and Western blotting analysis. α-Tubulin was used as a loading control. The scale bar represents 200 μm. EGFP, enhanced GFP; RHAU, RNA helicase associated with adenylate- and uridylate-rich element.

Previously, we figured out RHAU binding to the G4-forming sequences (G4S) of 5'-UTR in *Nkx2-5* mRNA to facilitate translation. Thus, we analyzed whether RHAU executes similar effects on the G4S in the 5'-UTRs of *Yap1* and *Hexim1* mRNAs. By sequencing analysis, potential G4-forming sequences (G4S) in both 5'-UTRs of *Yap1* and *Hexim1* mRNA were identified (Fig. 4*A*). We then constructed enhanced GFP (EGFP)–expressing vectors bearing the 5'-UTRs of *Yap1* and *Hexim1* (5'-UTR-EGFP) or their variants containing mutant G4S (G4Sm-EGFP) (Fig. 4, *A*–*B*). These vectors were then delivered into human embryonic kidney 293 (HEK293) cells. Afterward, the activity of GFP reporters was visualized by intensity and further examined by Western blotting analysis. Notably, the cells transfected with vectors bearing the 5'-UTRs (5'-UTR-EGFP) of *Yap1* or *Hexim1* mRNAs showed a dramatically reduced intensity of GFP compared with those cells transfected with a control vector (empty EGFP expression vector) (Fig. 4, *C*–*D*). However, the cells transfected with G4S mutant vectors (G4Sm-EGFP) showed comparable GFP intensity to those transfected with the control vector. Western blotting analysis further confirmed these changes (Fig. 4, *C*–*D*). Taken together, these results demonstrated that the G4S in the 5'-UTRs of *Yap1* and *Hexim1* mRNA strongly suppressed reporter activity.

Next, we explored whether RHAU could act on the G4S in the 5'-UTRs of *Yap1* and *Hexim1* mRNA to expedite translation. For this purpose, the activity of 5'-UTR-EGFP reporters was detected using strategy similar as described previously, with or without silencing of *Rhau*. The results showed that *Rhau* knockdown significantly reduced GFP reporter activity for both *Yap1* and *Hexim1* (Fig. 4, *E*–*F*). Conversely, *Rhau* overexpression remarkably enhanced GFP reporter activity for both *Yap1* and *Hexim1* (Fig. S9, *A*–*B*).

Furthermore, RNA pull-down analysis was conducted for probes consisting of the G4S mutated 5'-UTRs of *Yap1* and *Hexim1*. The results demonstrated that the association of RHAU with the *Yap1* and *Hexim1* 5'-UTRs was abolished when the G4S were mutated (Fig. 4*G*). Accordingly, modulation of RHAU protein levels by *Rhau* knockdown or overexpression showed little effects on GFP reporter expression when the G4Ss were mutated (Figs. 4, *H*–*I* and S9, *C*–*D*). In addition, we also detected the transcript levels of these GFP reporters by qRT-PCR analysis. However, the results showed that modulation of RHAU levels by *Rhau* knockdown or overexpression showed little effects on GFP transcript levels (Fig. S10, *A*–*D*).

Taken together, these results suggest that the association of RHAU with the 5'-UTRs of *Yap1* and *Hexim1* mRNA is responsible for the translational regulation; and the G4Ss are required for the association of RHAU with the 5'-UTRs of *Yap1* and *Hexim1* mRNA, and the regulatory effects.

### *Rhau* deletion impairs heart regeneration in the neonatal mice

YAP1 is believed to be essential for neonatal heart regeneration in response to injury (26, 33, 34). In this study, we identified *Yap1*, *Hexim1*, and *Nkx2-5* mRNAs as a regulatory target of RHAU, and we found that *Rhau* deficiency profoundly reduced their protein levels. Therefore, we explored the capacity for heart regeneration in *Rhau*-deficient neonatal mice.

**Figure 5:**
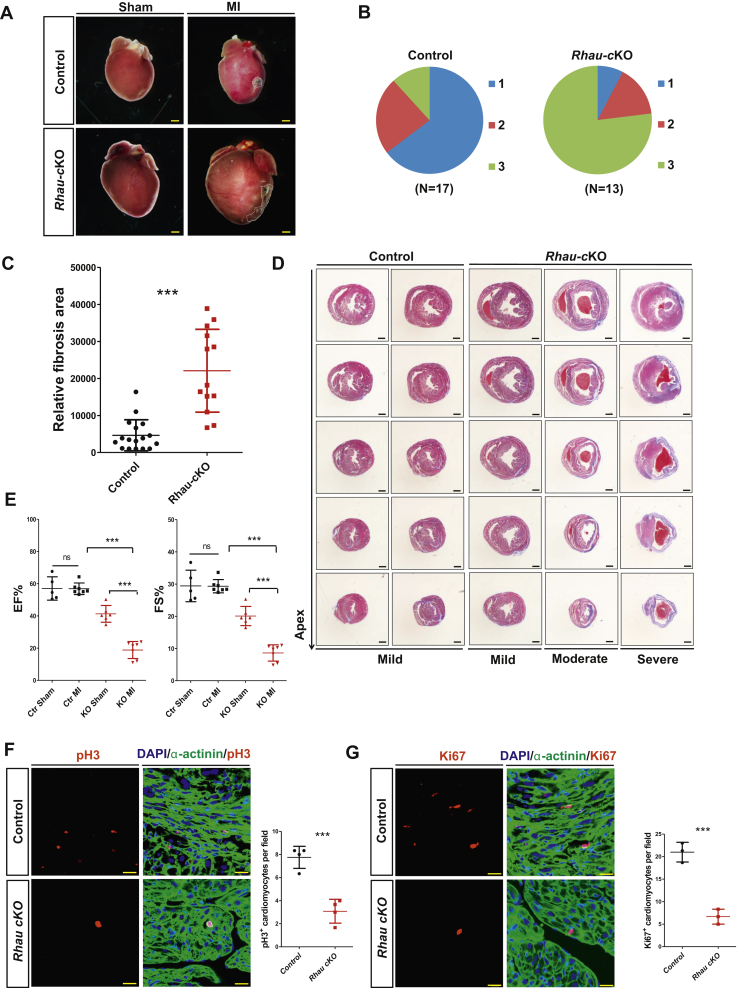
***Rhau* deletion impairs heart regeneration in the neonatal mice.***A*, representative images from morphological analysis of control (*Rhau*^*F/F*^) and *Rhau*-cKO (*α-MHC-Cre;Rhau*^*F/F*^) mice after MI surgery or a sham operation. Four weeks after MI surgery or sham operation (performed at P5), the hearts were collected for analysis. The scale bar represents 0.5 mm. *B*, heart regeneration capability scoring of control (*Rhau*^*F/F*^) mice (n = 17) and *Rhau*-cKO (*α-MHC-Cre;Rhau*^*F/F*^) mice (n = 13). The regeneration capability was grouped into three categories: category 1 indicated complete regeneration, category 2 represented partial regeneration, and category 3 indicated a total blockade of regeneration. *C*, quantification of fibrotic scar areas for control (*Rhau*^*F/F*^) mice (n = 17) and *Rhau*-cKO (*α-MHC-Cre;Rhau*^*F/F*^) mice (n = 13) 4 weeks after MI surgery. *D*, representative images from Masson's trichrome staining of heart sections for control (*Rhau*^*F/F*^) mice and *Rhau*-cKO (*α-MHC-Cre;Rhau*^*F/F*^) mice 4 weeks after MI surgery. The scale bar represents 0.5 mm. *E*, Echocardiography tests to examine the cardiac function of control (*Rhau*^*F/F*^) mice and *Rhau*-cKO (*α-MHC-Cre;Rhau*^*F/F*^) mice after MI or sham operation. Four weeks after MI surgery or sham operation (performed at P5), the mice were used for analysis. EF and FS were calculated according to the guidelines. For sham group, n = 5 and n = 6 for control and *Rhau*-cKO mice, respectively; for MI group, n = 7 for control or *Rhau*-cKO mice. *F*–*G*, immunofluorescence staining of pH3 (*F*) (n = 4) and Ki67 (*G*) (n = 3) labeled the proliferating cardiomyocytes after MI surgery and data quantification. One week after MI surgery performed at P5, the hearts of control (*Rhau*^*F/F*^) mice and *Rhau*-cKO (*α-MHC-Cre;Rhau*^*F/F*^) mice were collected for analysis. pH3 and Ki67 marked the proliferating cells, α-actinin marked the cardiomyocytes, and DAPI marked all the nuclei. The average number of proliferating cardiomyocytes from three fields per heart section was used for quantification. The scale bar represents 200 μm. DAPI, 4′,6-diamidino-2-phenylindole; EF, ejection fraction; FS, fractional shortening; MI, myocardial infarction; ns, not significant.

Myocardial infarction (MI) surgery was performed on *Rhau*-cKO mice and control mice at P5, when the mouse heart is capable of complete regeneration. Hearts were collected 4 weeks after MI surgery. The capacity for regeneration was evaluated by fibrosis area and unbiased regeneration scoring as reported before (35). The scoring of regeneration capability was grouped into three categories: category 1 indicated complete regeneration, category 2 represented partial regeneration, and category 3 marked a total blockade of regeneration. Histological analysis and statistical study of the regeneration scores revealed that the hearts of most (account for 64.70%) of control mice could fully regenerate within 4 weeks after MI (Fig. 5, *A*–*B*). However, a greater number (account for 76.92%) of the hearts of *Rhau*-cKO mice lost their regeneration capacity (Fig. 5, *A*–*B*). Moreover, the hearts of *Rhau*-cKO mice displayed markedly enlarged size after MI surgery compared with those of sham *Rhau* cKO mice (Fig. 5*A*).

Examination of the fibrotic scar areas revealed extensive fibrosis in the hearts of *Rhau*-cKO mice and little or no fibrosis in controls, which was consistent with the results concerning regeneration capacity (Fig. 5*C*). Masson's trichrome staining of heart sections further confirmed these results (Fig. 5*D*).

In addition, echocardiography was performed to examine the cardiac function of these mice. By 4 weeks after MI injury, the control mice had completely recovered their cardiac contractile function to the same level as the sham group (Fig. 5*E*). However, the cardiac contractile function was dramatically decreased in the MI group compared with the sham group in *Rhau*-cKO mice, which indicated a complete blockade of recovery (Fig. 5*E*).

To further establish the effects of RHAU on heart regeneration, we conducted immunofluorescent staining of phosphohistone H3 and Ki67 to label the proliferating cardiomyocytes before and after MI surgery. RHAU deficiency significantly reduced the number of phosphohistone H3 and Ki67 positive cardiomyocytes in *Rhau*-cKO mice hearts compared with those in control mice hearts (Fig. 5, *F–G*).

## Discussion

In this study, we have uncovered the essential role of RHAU in sustaining postnatal heart function. Deletion of *Rhau* in the postnatal cardiomyocytes imposes a profound effect on heart physiology. Heart contractility is impaired and quickly deteriorated leading to significant ventricular dilation and heart failure. Eventually, all the mice are lost within half a year. Thus, RHAU is essential for postnatal heart physiology. Meanwhile, this study suggests that RHAU deficiency could be one of the reasons in the development of DCM. This work unraveled the contribution of RHAUs to neonatal heart regeneration. Thus, modulation of RHAU amount could be a therapeutic strategy to interfere with a loss of cardiomyocytes and cardiac fibrosis resulting from DCM and MI.

**Figure 6:**
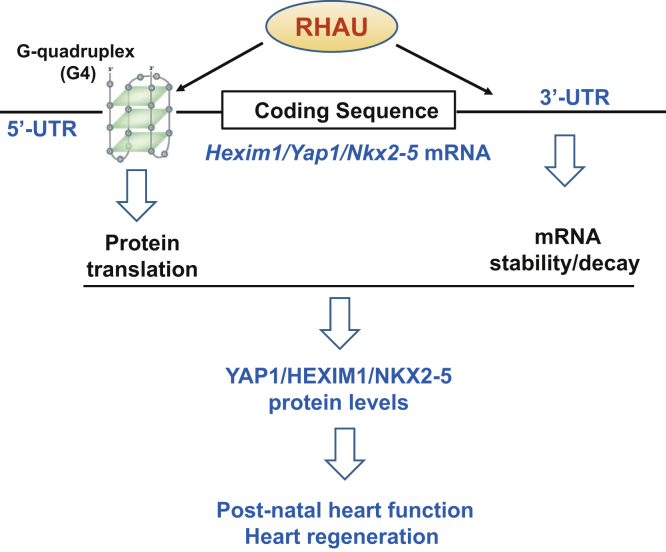
Working model of RHAU sustains postnatal heart function and neonatal regeneration through modulating target mRNA molecules. RHAU associates with both the 5'- and 3'-UTRs of the mRNA molecules of *Yap1*, *Hexim1*, and *Nkx2-5* to regulate mRNA translation and stability. The proteins of *YAP1*, *HEXIM1*, and *NKX2-5* are important regulators for heart function and regeneration. RHAU, RNA helicase associated with adenylate- and uridylate-rich element.

RHAU is an mRBP with G4 resolvase activity (14, 17). The results presented in this study, together with our previous investigation, point to a unique dual regulatory mechanism exerted by RHAU to modulate mRNA translation and stability in a structure- and sequence-specific/dependent manner. RHAU acts on the G4 structures in the 5'-UTRs of *Yap1*, *Hexim1*, and *Nkx2-5* mRNAs to facilitate translation, whereas its association with the 3′-UTR of these mRNAs promotes their decay (Fig. 6). The important functions of YAP1, HEXIM1, and NKX2-5 in heart development and postnatal heart function have been comprehensively investigated and confirmed in the past; this study, therefore, suggests that RHAU sustains postnatal heart function and neonatal heart regeneration through a collective action of these proteins (Fig. 6).

The G4 sequence/structure can be found in a wide range of locations in mRNA molecules, with preference for the 5'- and 3'-UTRs (36, 37). More than 13,000 rG4s (RNA G4s) forming sequences have been identified by *in vitro* and *in silico* approaches in the human transcriptome with the potential to form rG4s. A recent study mapped globally RHAU (DHX36) binding to RNA in human cells and revealed more than 4500 mRNAs possessing G-rich and G4s (38, 39, 40). To date, four RNA helicases have been suggested to unwind the G4 structures in mRNAs to facilitate translation; these helicases are RHAU/DHX36, DHX9, DDX21, and eIF4A although direct evidence for eIF4A to resolve G4 motif is still lacking (41). A recent study has reported that RHAU expedites the translation of *Mll1* and *Mll4* mRNAs *via* binding to the regions harboring the G4 motifs (42). eIF4A plays an essential role in translation initiation and has been shown to promote the translation of multiple oncogenic mRNAs in a G4-motif–dependent manner, which contributes profoundly to tumorigenesis (41). Taken together with our work, these studies have presented convincing evidence that the G4 structure of mRNA molecules regulate translation, which is involved in organogenesis and physiology.

In summary, we have demonstrated the important function of RHAU in regulating mRNA translation and stability, which is unique and vital for heart physiology. In the future, it will be necessary to clarify the details and sequential events of RHAU-mediated dual regulation of mRNA translation and degradation.

## Experimental procedures

### Mouse models and ethics statement

The experimental animal facility has been accredited by the Association for Assessment and Accreditation of Laboratory Animal Care International. All mouse experiments were carried out according to the protocol approved by the Institutional Animal Care and Use Committee of the Model Animal Research Center of Nanjing University. Mice were anesthetized by inhalation of 1.0% isoflurane (RWD Life Science, China) in oxygen and placed on a warming pad during the whole process for echocardiographic measurement. All mice used in this study were bred on a C57BL/6J background.

*Rhau* floxed (*Rhau*^F/F^) mice were generated as reported previously (19). To generate *Rhau* conditional knockout (*Rhau*-cKO) mice, we crossed *α-MHC-Cre* and *Rhau*^F/F^ mice, where *α-MHC-Cre* was always introduced from the male mouse. For chemically inducible deletion of *Rhau* in the adults, we crossed *Rhau*^*F/F*^ mice with the *CAAG-ERT2-Cre* (*ERT2-Cre*) line to generate *ERT2-Cre;Rhau*^*F/F*^ mice. On tamoxifen (Sigma, T5648) administration (intraperitoneal injection at 20 mg/kg per day for 5 days) at 2 months of age, *Rhau* was effectively deleted in these mice (*ERT2-cre;Rhau*^*F/F*^). Littermate controls (*Rhau*^*F/F*^) were used for all these comparisons. PCR primers for genotyping of Cre and *Rhau* floxed (*Rhau*^F/F^) mice are following: Cre forward, ATGCTTCTGTCCGTTTGC; Cre reverse, CAATATGGATTAACATTCTCCC; *Rhau* flox forward, CTGCGTAGGGTAGCTTATG; *Rhau* flox reverse, ATCCGACTGTAGATTCCTTT.

### MI models for heart repair and regeneration

MI surgery was performed as described previously (26, 43). Briefly, mice (P5) were anesthetized by cooling on an ice bed for 3 to 5 min. After a skin incision was made, the intercostal muscles were moved aside by blunt dissection at the fourth intercostal space. A 10-0 nonabsorbable suture was then passed through the midventricle below the origin of the left anterior descending coronary artery and tied off. Afterward, the ribs were sutured together with a 6-0 nonabsorbable suture, and the skin wound was closed by using skin glue. After surgery, the neonates were warmed rapidly for several minutes under a heating lamp until recovery. The entire procedure lasted for 10 min. Sham pups underwent the same procedure including hypothermic anesthesia and thoracotomy except for left anterior descending coronary artery ligation. To evaluate the capacity for heart regeneration, we first performed echocardiography analysis as described previously at 4 weeks after MI. The mice were then sacrificed for further analysis of fibrotic area and unbiased regeneration scoring.

### Echocardiography

Transthoracic echocardiography was performed by using a Vevo 770 UBM system (Visual-Sonics, Canada) equipped with a 30 MHz single-element mechanical transducer as described previously (44). Mice were anesthetized by inhalation of 1.0% isoflurane in oxygen and placed on a warming pad during the whole process. The body temperature of the mice was monitored and maintained between 36 and 38 °C. The heart rate was maintained between 350 and 450 beats/min. Two-dimensional guided M-mode tracings were recorded. After measurement, cardiac output values, such as ejection fraction, fractional shortening, and left ventricular internal diameter at the end of diastole, were calculated according to the guidelines accompanying the Vevo 770 UBM system. All these measurements were performed by an investigator who was blinded to the genotypes of the mice.

### Western blotting analysis

Western blotting was performed following standard procedures. Briefly, cell and/or tissue lysates were prepared in radioimmunoprecipitation assay buffer. After denaturation, samples were resolved in 10% SDS-PAGE gels and transferred onto polyvinylidene fluoride membranes (Millipore). The membranes were blocked with 5% nonfat milk in 50 mM Tris, 150 mM NaCl, 0.5 mM TWEEN 20, pH 7.5 for 1 h at room temperature. After being blocked, the membranes were incubated with primary antibodies overnight at 4 °C, followed by incubation with the horseradish peroxidase–conjugated secondary antibody for 2 h at room temperature. Tanon High-sig ECL Western Blotting Substrate (Tanon, China) was used to detect horseradish peroxidase activity, and the bands were visualized with the Tanon-5200 imaging system (Tanon, China). The antibodies used are provided in Table S3. Relative gray intensity is quantified by Adobe Photoshop CS5 (Adobe Inc., Fig. S11).

### Quantitative RT-PCR

Total cellular RNA was extracted using TRIzol Reagent (Life Technologies, 15596018) following the manufacturer's instructions. A reverse transcription reaction was performed to produce complementary DNAs by using a HiScript II Q RT Supermix for qPCR (+gDNA wiper) kit (Vazyme Biotech, R223). The complementary DNAs were subjected to quantitative real-time PCR (qRT-PCR) using an AceQ qPCR SYBR Green Master Mix Kit (Vazyme Biotech, Q111) and an ABI StepOne Plus Real-time PCR instrument (Applied Biosystems). Relative quantification of gene expression was based on the threshold cycle (Ct), with *Gapdh* or β-actin mRNA as the internal control. Data were normalized to a control value of 1 and presented as a fold change (FC). The primers used for qRT-PCR are provided in Table S4.

### Plasmid construction

The vector expressing RHAU was as described previously (21). For the construction of pGL3-Luc–derived reporter plasmids, the fragments containing the 5'-UTRs, CDSs, and 3'-UTRs of *Yap1* and *Hexim1* mRNAs were first amplified by PCR. The fragments were then inserted into the pGL3-Promoter vector (Promega, E1761), flanked by luciferase CDSs, using a high-efficiency ClonExpress One Step Cloning Kit (Vazyme Biotech, C112). The pEGFP-derived reporter plasmids containing the 5'-UTRs of *Yap1* and *Hexim1* mRNAs were cloned into pEGFP-N1 vector (Clontech, 6085-1) using a similar strategy. As to 5'-UTRs G4S-mutated EGFP reporter plasmids, the G4S-mutated templates were generated by overlapping PCR with mutation primers and subsequently inserted flanking the EGFP CDS. The primers for plasmid construction are listed in Table S5.

### Cell culture and transfection

H9C2 and HEK293 cells were maintained in DMEM (Gibco, 11995065), supplemented with 10% fetal calf serum (Gibco, 10099141), at 37 °C in the presence of 5% carbon dioxide. Transient transfection was performed by using Lipofectamine 3000 (Life Technologies, L3000015) according to the manufacturer's instructions. To silence *Rhau*, siRNAs targeting *Rhau* mRNAs were transfected into the H9C2 or HEK293 cells. Control cells were transfected with a negative control siRNA. The sequences for the siRNAs are listed in Table S6. Cells were collected 72 h after siRNA transfection for further analysis. For plasmid transfection, cells were collected 48 h after transfection for further analysis.

### Luciferase assay

A firefly luciferase reporter assay was conducted according to the protocol from the manufacturer (Dual-Luciferase Reporter Assay System, Promega, E1910). For the luciferase reporter assay with *Rhau* knockdown, H9C2 cells were seeded in 6 cm dishes and initially transfected with siRNA targeting *Rhau* mRNA (or negative controls) as described previously. Twenty-four hours later, cells were reseeded in 24-well plates (4 × 10^4^ cells per well). The reseeded cells were subsequently transfected with 100 ng of pGL3-reporter (containing 5'-UTRs, CDSs, and 3'-UTRs of *Yap1* and *Hexim1* mRNAs) plasmids or blank pGL3-reporter (pGL3-Promoter) plasmid. Meanwhile, 0.5 ng of pRL-TK *Renilla* reporter plasmid (Promega, E2241) was transfected as internal controls for each group. Seventy-two hours after the siRNA transfection, cell lysates were prepared to measure the transcripts levels by qRT-PCR or luciferase activity following the manufacturer's instructions. Data were normalized to a control value of 1 and presented as an FC.

### RNA pull-down analysis

RNA pull-down assays were performed as described previously (45). To transcribe biotinylated RNA probes, we used PCR-amplified DNA fragments of 5'-UTRs, CDSs, and 3'-UTRs with the T7 promoter (CCAAGCTTCTAATACGACTCACTATAGGGAGA) as templates. The probes were then produced by using T7 RNA polymerase with biotin-uridine triphosphate (Biotium, #40033). One microgram of purified biotinylated transcripts was then incubated with 200 μg of whole-cell H9C2 lysates for 30 min at room temperature. Afterward, the washed Dynabeads M-280 Streptavidin (Life Technologies, 11206D) were added to the complexes for a further incubation of 30 min. The complexes were isolated using a magnetic grate (Life Technologies, 12321D) and washed with cold PBS three times. Finally, the pull-down material was analyzed by Western blotting analysis as described previously. Primers used for production of biotinylated RNA probes are listed in Table S7.

### RIP assay

RIP was performed using H9C2 cells with RHAU antibody as described previously (21). In brief, H9C2 cells were exposed to ultraviolet C (300 mJ/cm^2^), and the whole-cell lysates were collected for immunoprecipitation. The prepared lysates were then incubated with RHAU antibody (or immunoglobulin G antibody acted as control) for 2 h at 4 °C, followed by an additional 2 h of incubation with Protein G beads (Life Technologies, 10004D). The beads were washed five times with lysis buffer, and RNA was extracted by addition of 1 ml of TRIzol to the beads. Afterward, the extracted RNA was dissolved in 20 μl of RNase-free water and further analyzed by qPT-PCR. The reverse transcription reaction and qRT-PCR analysis were performed following the same procedures as described previously.

### Half-life analysis of mRNAs

To analysis the half-life of endogenous *Yap1* and *Hexim1* mRNAs, H9C2 cells were transfected with siRNA targeting *Rhau* mRNAs or negative control siRNA. Forty-eight hours later, cells were reseeded, and 2 μg/ml actinomycin D (Amresco, AJ608) was added to the H9C2 cell culture medium. Total RNA was extracted at the times indicated (0, 2, 4, 6, and 8 h) and subjected to qRT-PCR analysis using specific primers.

### Histological analysis and immunostaining

Histological analysis was performed as described previously (21). Briefly, hearts were dissected, washed with PBS, and fixed in 4% paraformaldehyde overnight at 4 °C. The samples were then dehydrated and embedded in paraffin for preparation of 7 μm histological sections. Afterward, the rehydrated slides were stained with hematoxylin and eosin, Masson's stain, or used for TUNEL (Roche, 12156792910), immunofluorescence, and immunohistochemical analysis. Images were processed with an Olympus DP2-BSW microscope. To calculate the proportion of cardiac collagen deposition/interstitial fibrosis, we divided the total area of collagen by the total area of the left ventricle and then multiplied the result by 100%.

For immunostaining, frozen sections were washed three times with PBS and blocked in 10% goat serum (Boster Biological Technology, China) for 1 h at room temperature. The sections were then incubated with primary antibodies (overnight at 4 °C), followed by incubation with secondary antibodies (at room temperature for 2 h). During the secondary antibody incubation, 4',6-diamidino-2-phenylindole stain (Sangon biotech, D6584) was added to label the nucleus. Fluorescence images were taken with a 63× oil immersion objective on a Leica SP5 II confocal microscope system. The detailed information of the antibodies used in this study is provided in Table S3.

For determination of cardiomyocyte cross-sectional sizes, deparaffinized and rehydrated heart sections were incubated for 30 min at room temperature with Alexa Fluor 488–labeled wheat germ agglutinin (Thermo, W11261) to visualize myocyte membranes. To label the nucleus, 4',6-diamidino-2-phenylindole stain was also added. Regions of the left ventricular walls that included the circular shapes of cardiomyocytes were selected. The mean cross-sectional area of cardiomyocytes was determined from 60 to 80 cells. Data were normalized to a control value of 1 and presented as an FC.

### RNA-seq and bioinformatics analysis

Three hearts were dissected from control (*Rhau*^*F/F*^) and *Rhau*-cKO (*α-MHC-Cre;Rhau*^*F/F*^) mice at P10. Total RNA was isolated from the hearts by using TRIzol Teagent (Life Technologies, 15596018) following the manufacturer's instructions. The RNA samples were then sent to Vazyme Biotech (Nanjing, China) for further processing, including quality examination, library preparation, sequencing, and bioinformatics analysis.

#### RNA quantification and quality examination

RNA concentration was measured by using Qubit RNA Assay Kit (Life Technologies, Q32855) in Qubit 3.0 Fluorometer. The integrity of RNA samples was assessed by using the RNA Nano 6000 Assay Kit with the Bioanalyzer 2100 system (Agilent Technologies).

#### Library preparation and sequencing

A total amount of 1 μg of qualified RNA per sample was used as input material for the library preparation. The libraries for sequencing were generated by using the VAHTS mRNA-seq v2 Library Prep Kit for Illumina (Vazyme Biotech, NR601) following manufacturer's recommendations. Library concentration was measured by using Qubit RNA Assay Kit in Qubit 3.0 Fluorometer to preliminary quantify. After clustering of index-coded samples, the library preparations were sequenced on an Illumina HiSeq X Ten platform and 150 bp paired-end module.

#### Bioinformatics analysis

All the following analyses were based on clean data (6G) with high quality. The mapped clean reads of each sample were assembled using Cufflinks (v2.2.1) as previously reported (46). Fragments per kilobase of exon per million fragments mapped were calculated for coding genes. Cuffdiff (v2.2.1) provides statistical routines for determining differential expression in digital transcript or gene expression data sets. Genes with *p* < 0.05 and the absolute value of FC ≥1.2 were classified as significantly differentially expressed. Gene expression patterns were then depicted as a heatmap through the pheatmap package. GO enrichment analysis of differentially expressed genes was implemented with a Perl module (GO::TermFinder) (47). GO terms with corrected *p* values less than 0.05 were considered significantly enriched among the differentially expressed genes. R functions were used to test for the statistical enrichment of the differentially expressed genes among the KEGG pathways.

### Microarray analysis

For microarray analysis, hearts were collected at 2 months after tamoxifen treatment of adult *ERT2-cre;Rhau*^*F/F*^ mice and controls (*Rhau*^*F/F*^). Total RNA was extracted using TRIZOL Reagent as described previously, and RNA was assessed with an Agilent Bioanalyzer 2100 (Agilent technologies) to measure RNA integrity. Qualified total RNA was further purified with an RNeasy Micro Kit (QIAGEN, #74004) and an RNase-Free DNase Set (QIAGEN, #79254). Afterward, total RNA was amplified, labeled, and purified by using a GeneChip 3′IVT Express Kit (Affymetrix, #901229) following the manufacturer's instructions to obtain biotin-labeled cRNA. Array hybridization and washing were performed using a GeneChip Hybridization, Wash and Stain Kit (Affymetrix, #900720) in Hybridization Oven 645 (Affymetrix) and a Fluidics Station 450 (Affymetrix) according to the manufacturer's instructions. Slides were scanned by a GeneChip Scanner 3000 (Affymetrix) and Command Console Software 3.1 (Affymetrix) with default settings. Raw data were normalized by the MAS 5.0 algorithm, Gene Spring Software 11.0 (Agilent Technologies). The data were analyzed by use of the eBioService System (Shanghai Biotechnology, China).

### Statistical analysis

Data analysis was performed using GraphPad Prism software, version 5.0. Quantifications were performed using ImageJ. Statistical comparisons were carried out using two-tailed Student *t* tests. A value of *p* < 0.05 (∗) was considered statistically significant, whereas *p* < 0.01 (∗∗) and *p* < 0.001 (∗∗∗) were considered very statistically significant, and not significant with *p* value ≥0.05 represented no statistical difference. Details on sample numbers and significance levels are given in the figure legends. In all figures, measurements are reported as the mean ± S.D.

## Data availability

All representative data are contained within the article. Microarray data have been deposited in Gene Expression Omnibus under accession number GSE119384. RNA-seq data are available on request from the corresponding author: Zhongzhou Yang (zhongzhouyang@nju.edu.cn), Nanjing University.

## Conflict of interest

The authors declare that they have no conflicts of interest with the contents of this article.
