# Supplementary material for: The G4 resolvase RHAU modulates mRNA translation and stability to sustain postnatal heart function and regeneration
Source: J Biol Chem. 2020 Nov 23;296:100080. doi: 10.1074/jbc.RA120.014948 (PMC7948451; doi:10.1074/jbc.RA120.014948)
Supplement: Supplementary Figures and Tables [file mmc1.pdf]

Supplemental Information

RHAU sustains post-natal heart function and regeneration through modulating mRNA translation and stability

Mingyang Jiang,[1,4] Han Hu,[2,4] Ke Zhao,[1] Ruomin Di,[3] Xinyi Huang,[1] Yingchao Shi,[1] Yunyun Yue,[1] Junwei Nie,[1] Shan Yu, [1] Wengong Wang,[2,*] and Zhongzhou Yang[1,*]

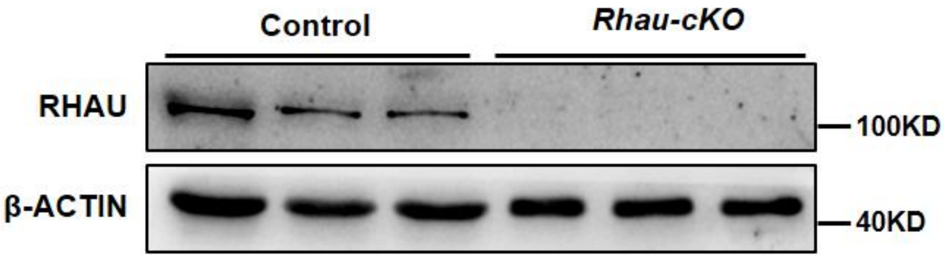

Figure S1. Western blotting analysis of RHAU protein levels in the isolated cardiomyocytes. Cardiomyocytes were isolated from *Rhau-cKO* and control mice at two months for Western blotting analysis to detect the protein levels of RHAU. β-ACTIN was used as loading controls.

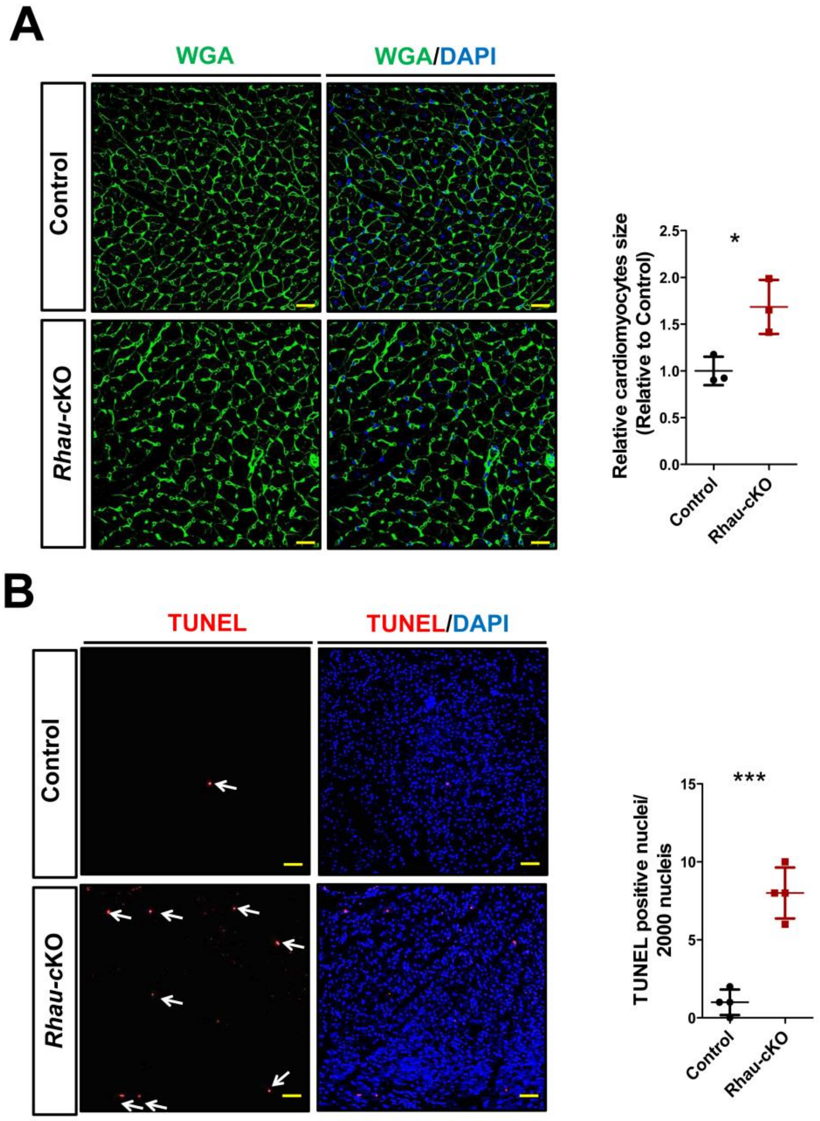

Figure S2. WGA and TUNEL staining of cardiomyocytes.
(A) WGA staining of cardiomyocytes size for *Rhau*-cKO (*α-MHC-Cre;Rhau*$^{F/F}$) and control (*Rhau*$^{F/F}$) hearts at 2 month old. The cardiomyocytes size was quantified using 60 to 80 cardiomyocytes. Data were normalized to control value of 1 and presented as a fold change. Scale bars, 50 µm. (B) TUNEL assay for cell apoptosis in the *Rhau*-cKO and control hearts. Three hearts from *Rhau*-cKO mice and another three hearts from controls mice were used for quantification. Scale bars, 200 µm.

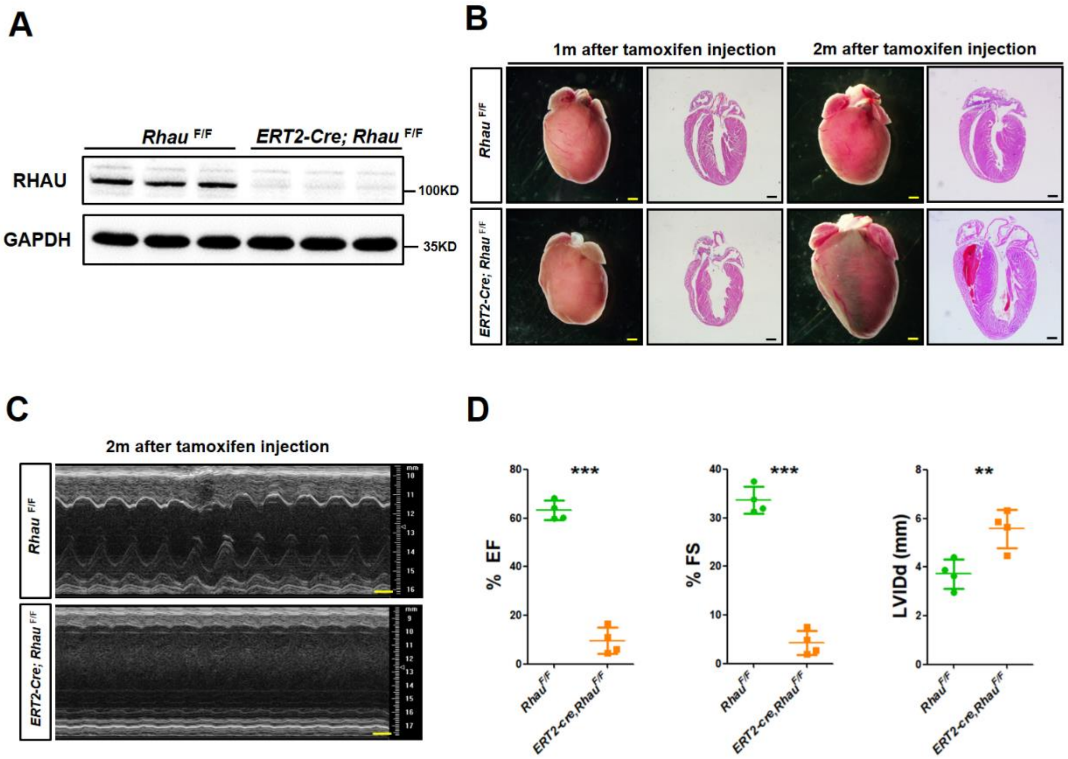

Figure S3. Morphological and echocardiographic analysis.
(A) Western blotting analysis to confirm the efficiency of *Rhau* deletion in the hearts of control (*Rhau*$^{F/F}$) mice and *ERT2-Cre;Rhau*$^{F/F}$ mice. GAPDH was used as loading controls. Tamoxifen (20 mg/kg/day) was administered by intraperitoneal injection for 5 days starting at 2 months age. Afterwards, the hearts were collected for Western blotting analysis. (B) Morphological and histological analysis of hearts from *Rhau*$^{F/F}$ and *ERT2-Cre;Rhau*$^{F/F}$ mice. Tamoxifen (20 mg/kg/day) was administered as described above. The hearts were collected at 1 month or 2 months after tamoxifen administration. Scale bars, 0.5 mm. (C) Representative images of M-mode echocardiography of *Rhau*$^{F/F}$ and *ERT2-Cre;Rhau*$^{F/F}$ mouse hearts at 2 months after tamoxifen administration. Scale bars, 1 mm. (D) Echocardiographic analysis of *Rhau*$^{F/F}$ mice and *ERT2-Cre;Rhau*$^{F/F}$ mice at 2 months after tamoxifen administration. EF (ejection fraction), FS (fractional shortening) and LVIDd (Left ventricular internal diameter at end-diastole) were calculated according to the guidelines accompanying the Vevo 770 UBM system. N=4 for each group.

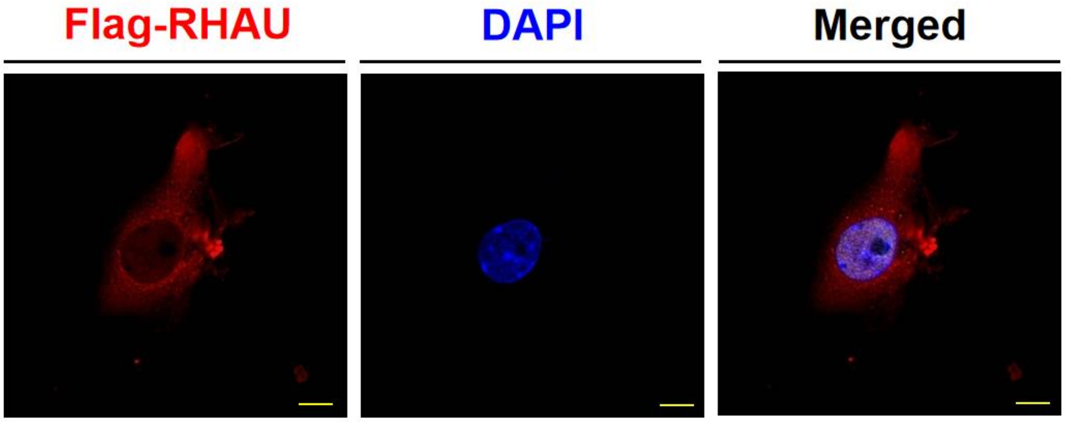

Figure S4. The localization of RHAU in the cardiomyocytes.
Flag-Rhau was overexpressed in cardiomyocytes. Afterwards, immunofluorescence staining was performed to detect Flag-RHAU.

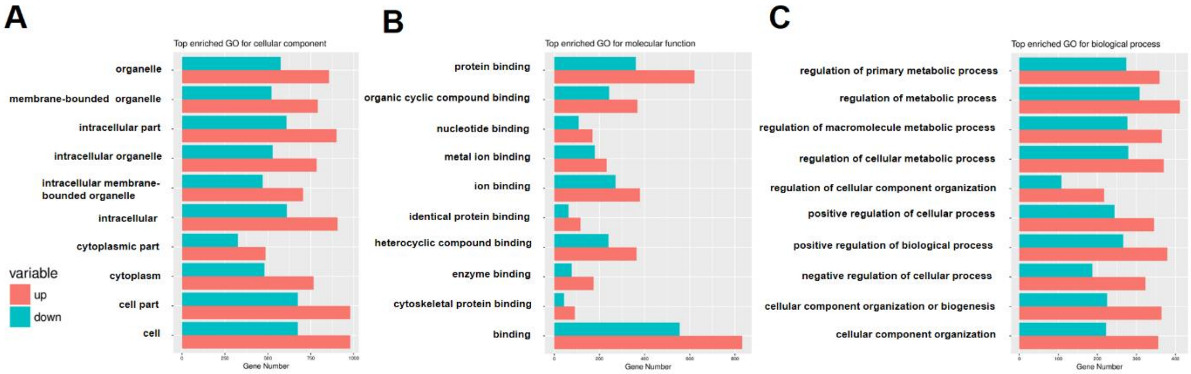

Figure S5. Gene ontology (GO) analysis to identify top enriched GO terms from RNA-Seq data.
Gene ontology (GO) analysis to identify enriched GO terms from RNA-Seq (P10) for cellular components (A), molecular functions (B) and biological processes (C).

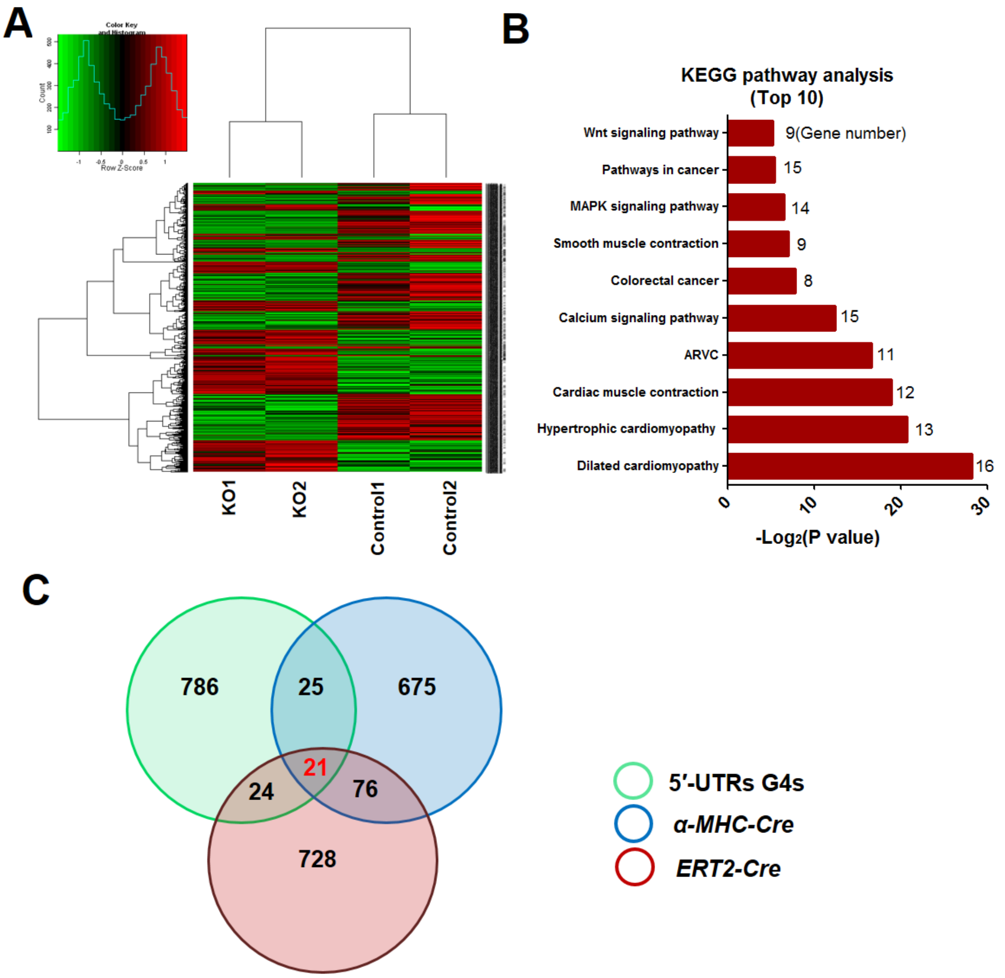

Figure S6. Heatmap and KEGG pathway analysis.

(A) Heatmap of microarray analysis for control (*Rhau*$^{F/F}$) and KO (*ERT2-Cre;Rhau*$^{F/F}$) mouse hearts at 2 months after tamoxifen administration. Differentially expressed genes were filtered with fold change ≥2 and *p*<0.05. (B) KEGG pathway analysis of differentially expressed genes from microarray data. (C) Venn diagram of gene numbers identified by RNA-Seq (*α-MHC-Cre*), microarrays (*ERT2-Cre*) and an mRNA 5'-UTRs G-quadruplexes (5'-UTRs G4s) database.

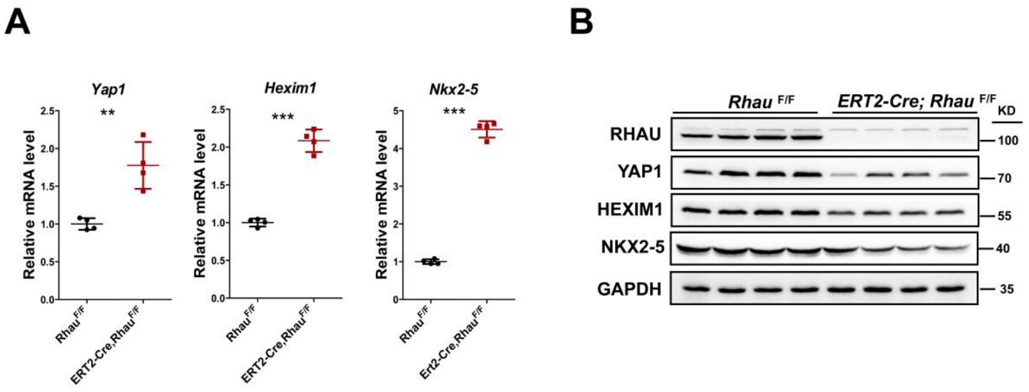

Figure S7. mRNA and protein level of YAP1, HEXIM1 and NKX2-5 in control (*Rhau*$^{F/F}$) and *ERT2-Cre;Rhau*$^{F/F}$ mouse hearts.

(A) Relative mRNA levels of *Yap1* and *Hexim1* in the hearts of control (*Rhau*$^{F/F}$) mice and *ERT2-Cre;Rhau*$^{F/F}$ mice were measured by qRT-PCR. Tamoxifen (20 mg/kg/day) was administered by intraperitoneal injection for 5 successive days starting at 2 months of age, and the hearts were then collected for qRT-PCR analysis. N=4 for each group. (B) Western blotting analysis was used to measure YAP1, HEXIM1 and NKX2-5 protein levels in the hearts of control (*Rhau*$^{F/F}$) mice and *ERT2-Cre;Rhau*$^{F/F}$ mice. GAPDH was used as loading controls. Tamoxifen (20 mg/kg/day) was administered by intraperitoneal injection for 5 days starting at 2 months age. Afterwards, the hearts were collected for Western blotting analysis.

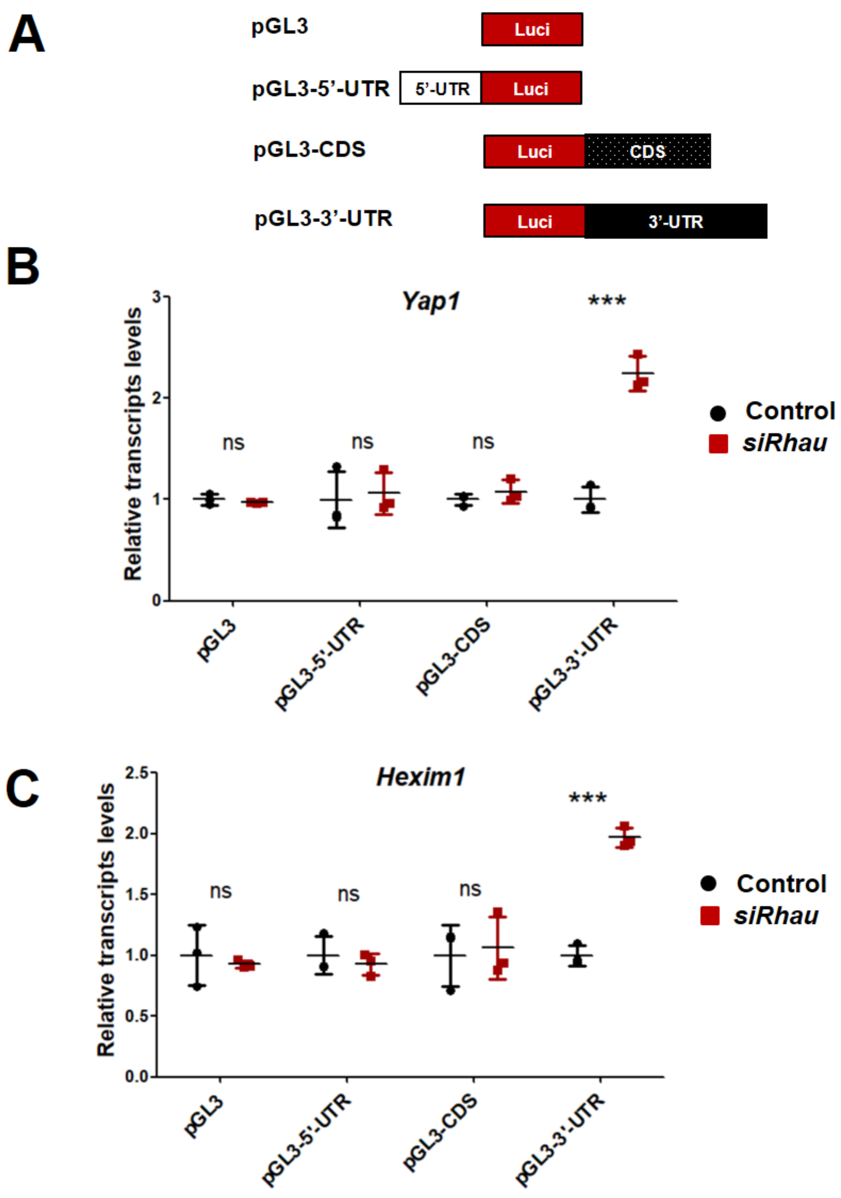

Figure S8. Luciferase reporter analysis.

(A) Schematic representation of the chimeric Luciferase vector structure. pGL3 represents pGL3-promoter blank vector; pGL3-5′-UTR represents chimeric pGL3-promoter vector flanked with 5′-UTR of *Yap1* or *Hexim1* mRNA; pGL3-CDS represents chimeric pGL3-promoter vector flanked with CDS (coding sequence) of *Yap1* or *Hexim1* mRNA; pGL3-3′-UTR represents pGL3-promoter vector flanked with 3′-UTR of *Yap1* or *Hexim1* mRNA. (B-C) qRT-PCR analysis to examine the chimeric luciferase transcripts levels of *Yap1* and *Hexim1* after *Rhau* knockdown in H9C2 cells. For each group, n=3.

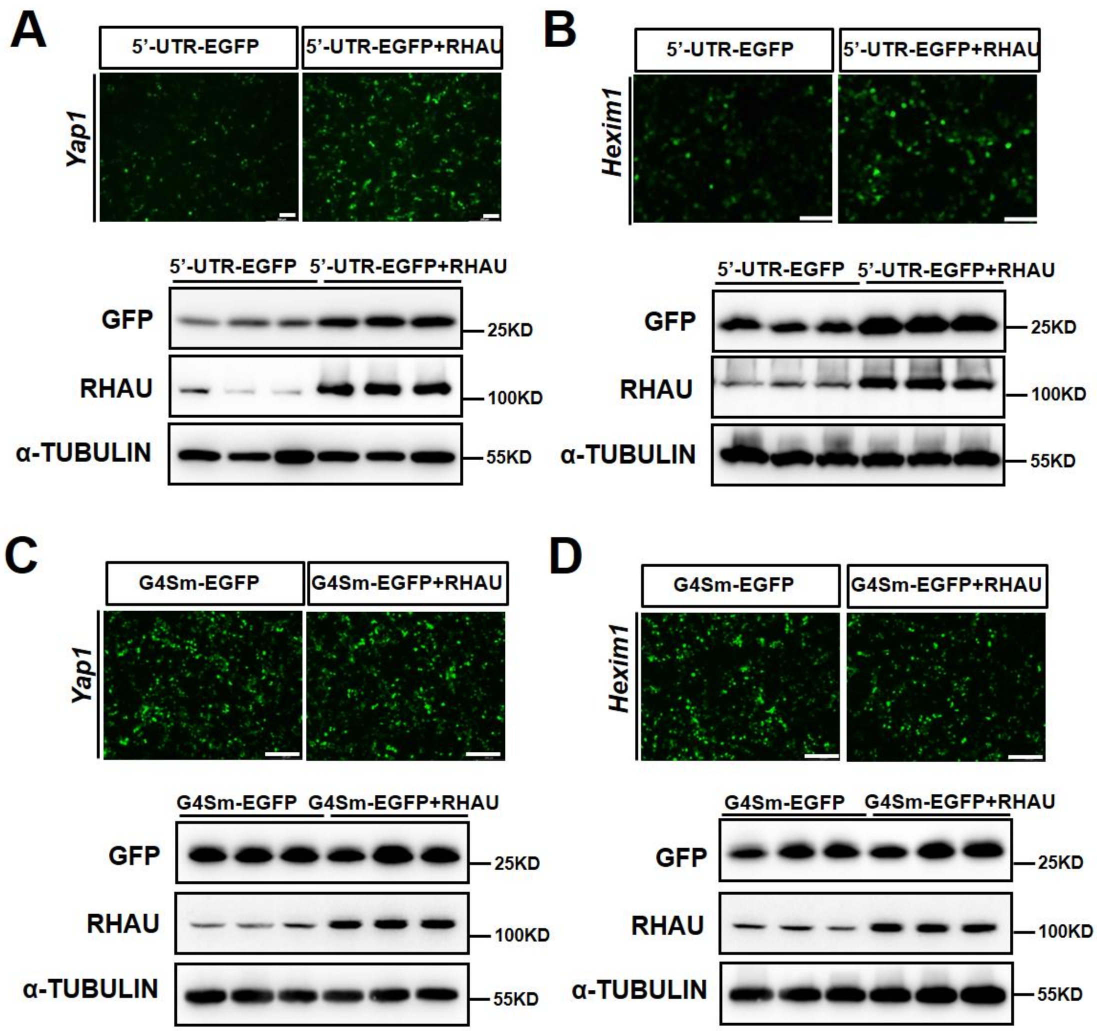

Figure S9. GFP reporter analysis.

(A-B) Representative images show that *Rhau* knockdown reduced the activity of chimeric GFP reporters for *Yap1* and *Hexim1* following by Western blotting analysis. α-TUBULIN was used as a loading control. Scale bars, 200 μm. (C-D) Representative images show the activity of G4Sm-EGFP reporters for *Yap1* and *Hexim1* in response to *Rhau* knockdown and Western blotting analysis. α-TUBULIN was used as a loading control. Scale bars, 200 μm.

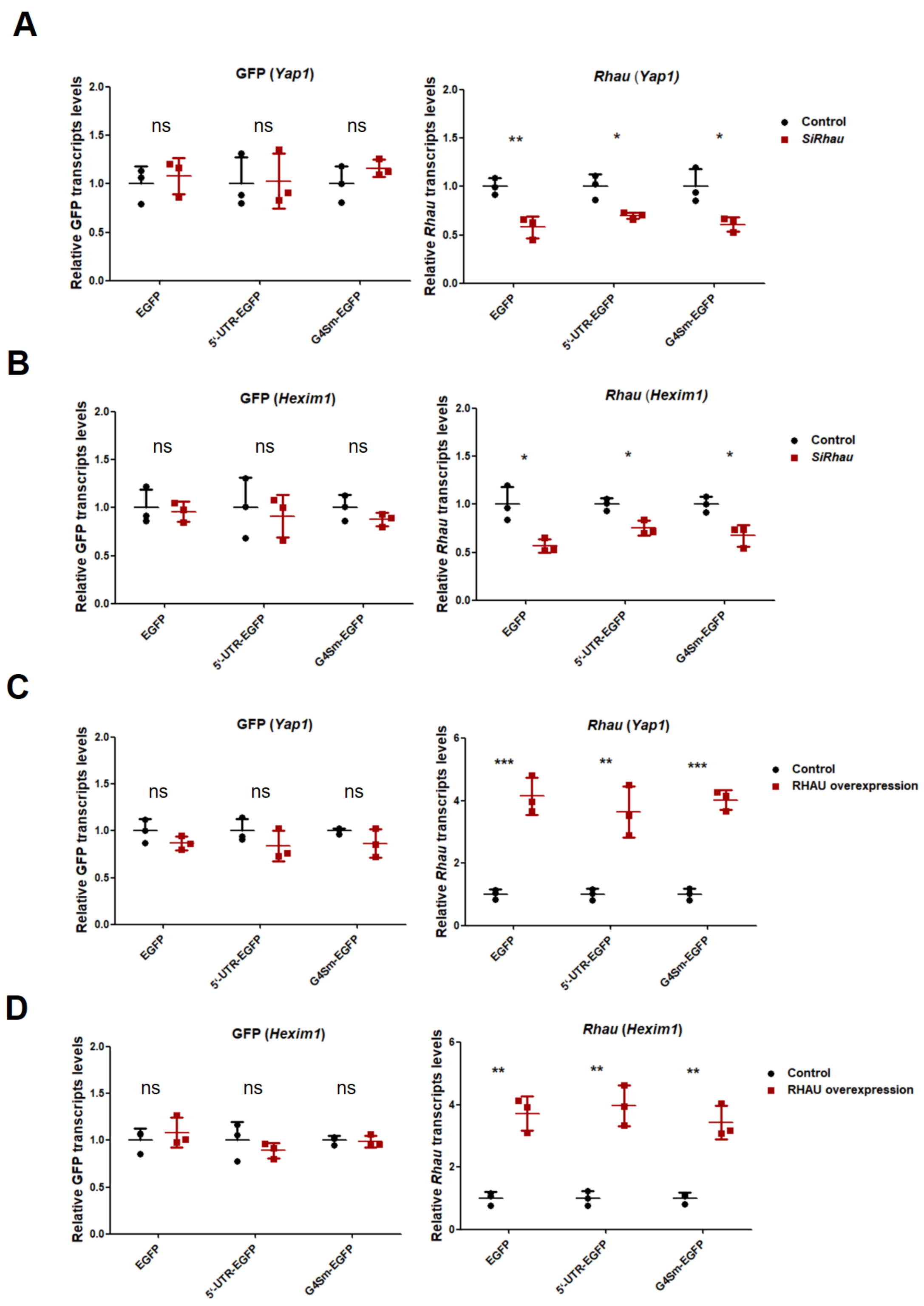

Figure S10. The transcripts levels of GFP reporter analysis.

(A-B) qRT-PCR analysis to examine the chimeric GFP reporter transcripts levels of *Yap1* (A) and *Hexim1* (B) after *Rhau* knockdown. The transfection assay was conducted as described in experimental procedures. Briefly, HEK293 cells were transfected with siRNA targeting *Rhau* mRNAs followed by chimeric GFP reporter transfection. Afterwards, the cells were

harvested and subjected to total RNA isolation followed by qRT-PCR analysis. The right panel confirmed the efficiency for *Rhau* knockdown. For each group, n=3. (C-D) qRT-PCR analysis to examine the chimeric GFP reporter transcripts levels of *Yap1* (C) and *Hexim1* (D) after *Rhau* overexpression. The transfection assay was conducted as described in experimental procedures. Briefly, HEK293 cells were co-transfected with *Rhau* overexpression plasmid and chimeric GFP reporters. Afterwards, the cells were harvested for qRT-PCR analysis. The right panel confirmed the efficiency for RHAU overexpression. For each group, n=3.

Figure S11. The gray intensity is quantified for Western Blotting ananlysis.

Figure S11-1 related to Figure 1A

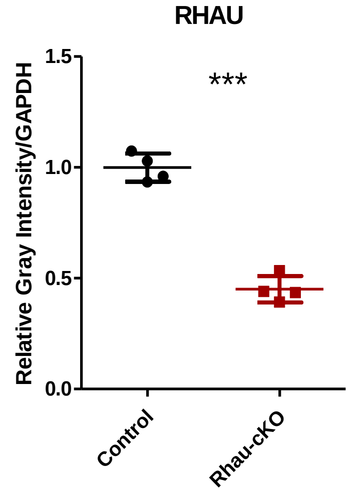

Figure S11-2 related to Figure 2D

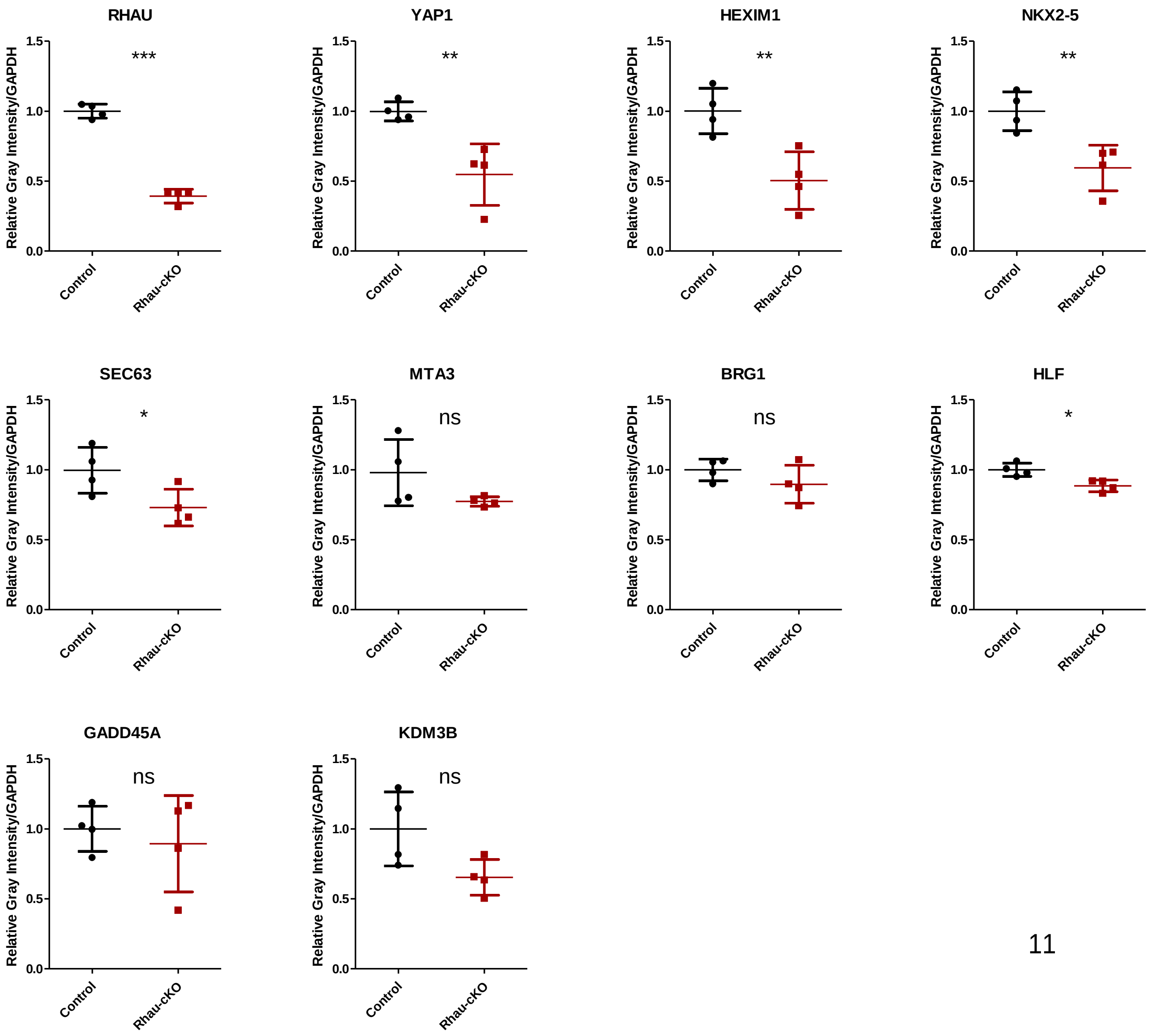

Figure S11-3 related to Figure 2F

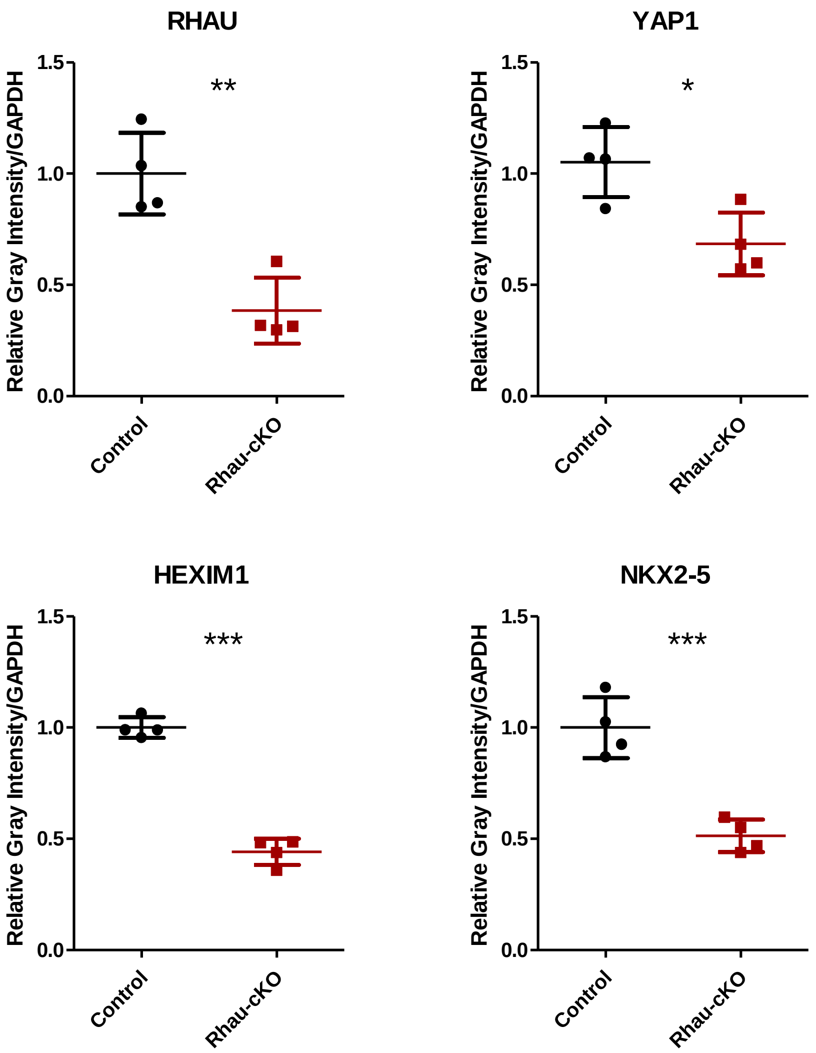

Figure S11-4 related to Figure 3B

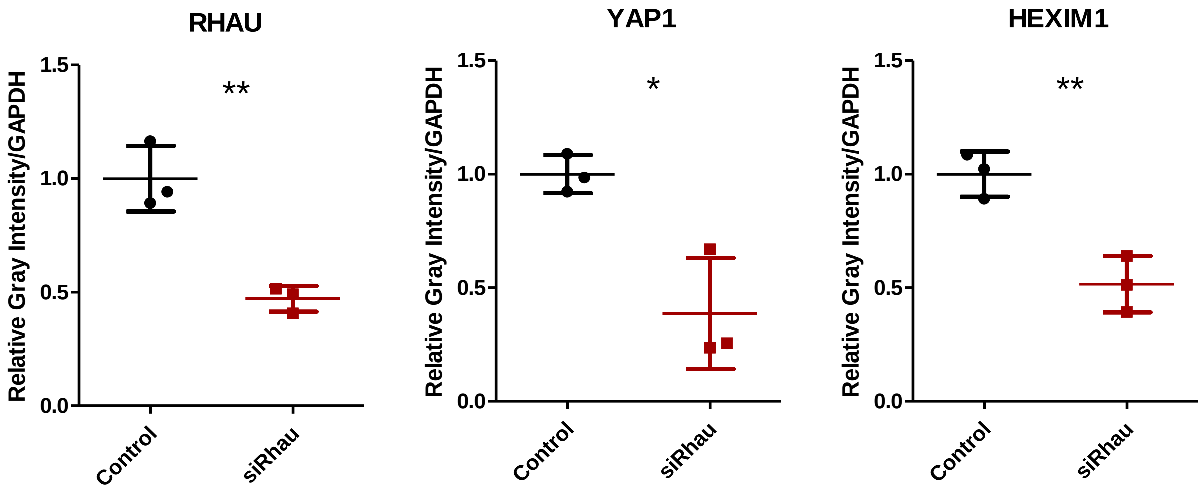

Figure S11-5 related to Figure 4C-4F, 4H-4I

(related to Figure 4C)

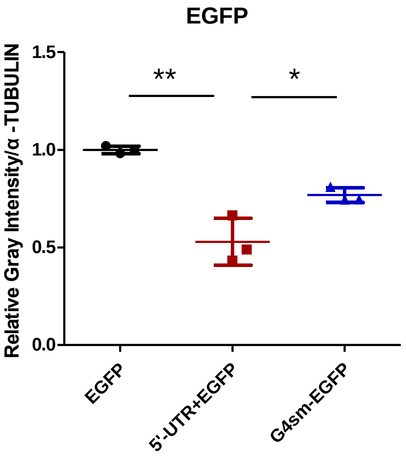

(related to Figure 4D)

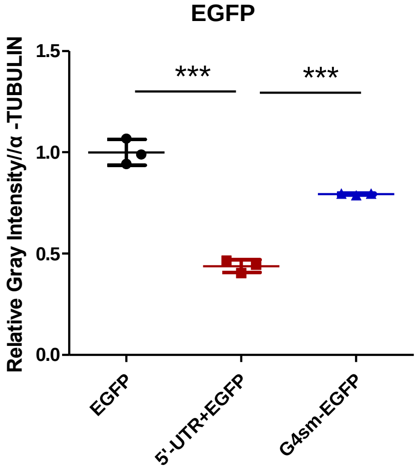

(related to Figure 4E)

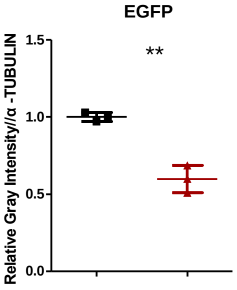

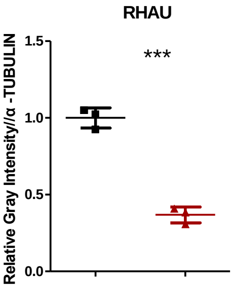

Yap1 5'-UTR-EGFP Control

Yap1 5'-UTR-EGFP *siRhau*

(related to Figure 4F)

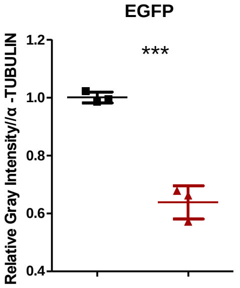

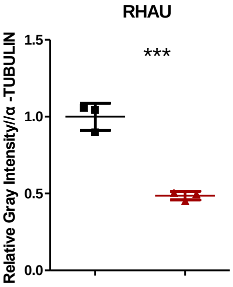

Hexim1 5'-UTR-EGFP Control

Hexim1 5'-UTR-EGFP *siRhau*

(related to Figure 4H)

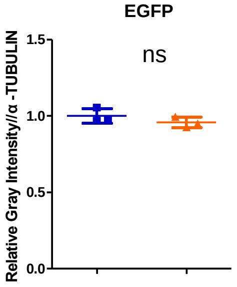

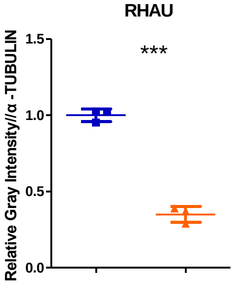

Yap1 G4sm-EGFP Control

Yap1 G4sm-EGFP *siRhau*

(related to Figure 4I)

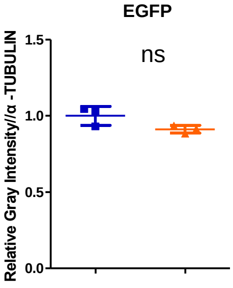

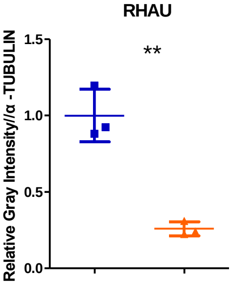

Hexim1 G4sm-EGFP Control

Hexim1 G4sm-EGFP *siRhau*

Figure S11-6 related to Figure S1

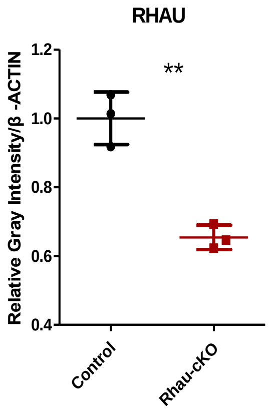

Figure S11-7 related to Figure S7B

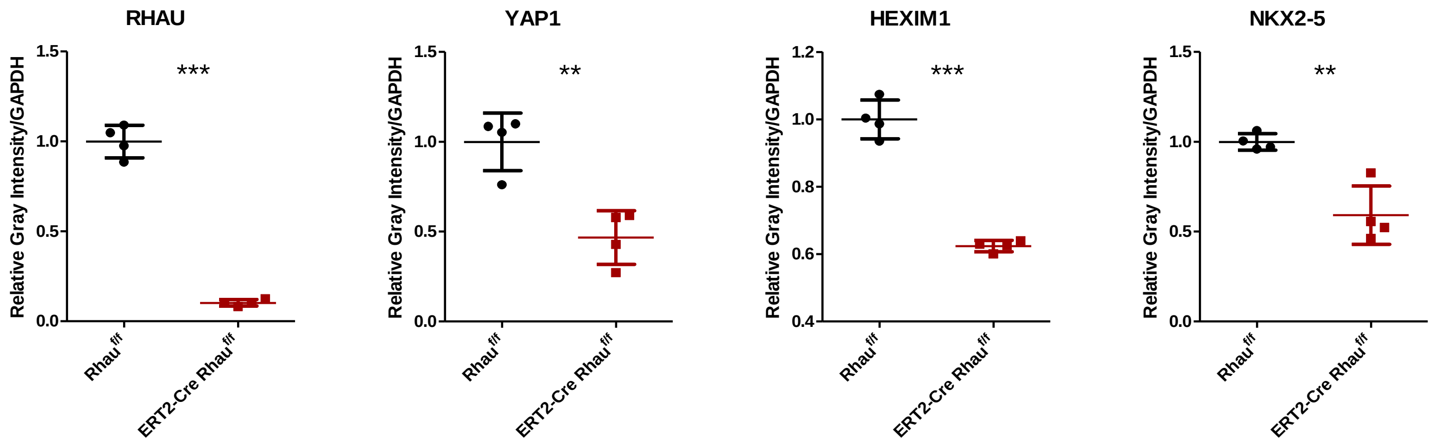

Figure S11-8 related to Figure S9

(related to Figure S9A)

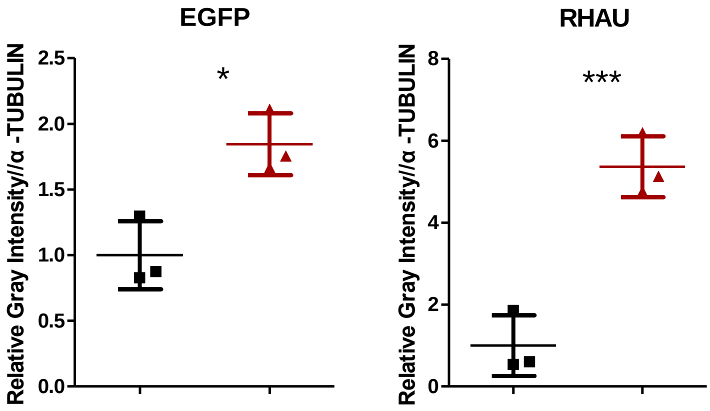

Yap1 5'-UTR-EGFP Control

Yap1 5'-UTR-EGFP RHAU

(related to Figure S9B)

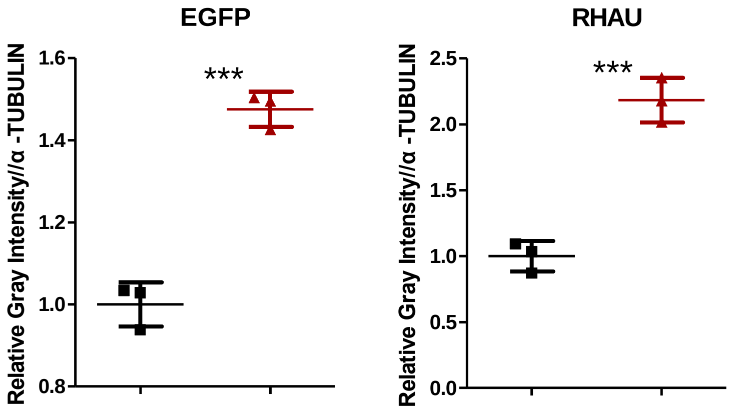

Hexim1 5'-UTR-EGFP Control

Hexim1 5'-UTR-EGFP RHAU

(related to Figure S9C)

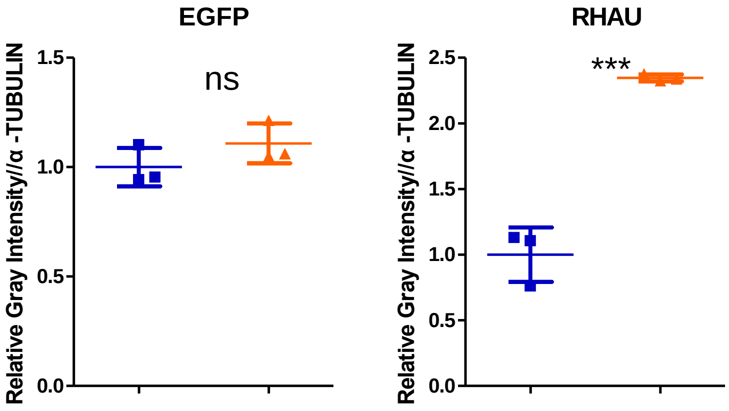

Yap1 G4sm-EGFP Control

Yap1 G4sm-EGFP RHAU

(related to Figure S9D)

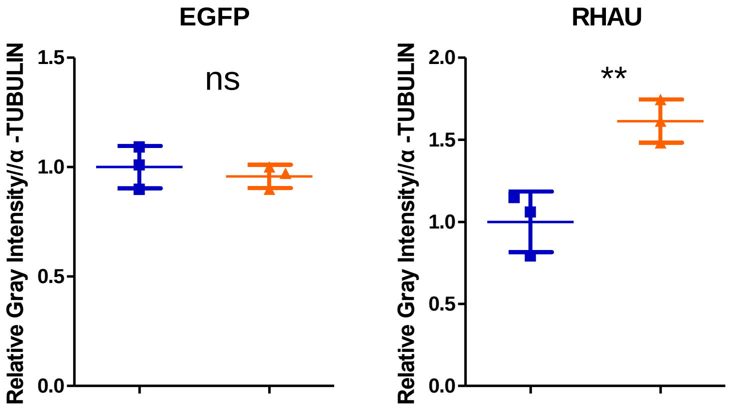

Hexim1 G4sm-EGFP Control

Hexim1 G4sm-EGFP RHAU

Table S1: Example genes for representative enriched signaling pathways.

Pathway	Gene Name	Fold Change of FPKM	up-or-down	p_value	Description
Dilated cardiomyopathy Pathway	*Adcy6*	1.553494356	up	5.00E-05	adenylate cyclase 6
	Itgb6	0.582105276	down	5.00E-05	integrin beta 6
	Myh7b	2.333782317	up	5.00E-05	myosin, heavy chain 7B, cardiac muscle, beta
	Cacna1s	2.784368194	up	0.01055	calcium channel, voltage-dependent, L type, alpha 1S subunit
Insulin signaling pathway	*Phka2*	0.614211317	down	0.00265	phosphorylase kinase alpha 2
	Phkg1	0.280462541	down	0.04435	phosphorylase kinase gamma 1
	Fbp2	0.581003194	down	5.00E-05	fructose bisphosphatase 2
	Mknk2	2.148530117	up	5.00E-05	MAP kinase-interacting serine/threonine kinase 2
	Inpp5j	1.567313737	up	5.00E-05	inositol polyphosphate 5-phosphatase J
	Flot1	1.681338257	up	5.00E-05	flotillin 1
Smooth muscle contraction pathway	*Mylk4*	0.500210068	down	5.00E-05	myosin light chain kinase family, member 4
	Prkg1	1.618959617	up	0.0027	protein kinase, cGMP-dependent, type I
	Adcy6	1.553494356	up	5.00E-05	adenylate cyclase 6
	Myl6	1.518580092	up	5.00E-05	myosin, light polypeptide 6, alkali, smooth muscle and non-muscle
	Adra1a	0.433078363	down	0.00145	adrenergic receptor, alpha 1a
	Myh7b	2.333782317	up	5.00E-05	myosin, heavy chain 7B, cardiac muscle, beta
	Myl9	1.838284422	up	5.00E-05	myosin, light polypeptide 9, regulatory
	Arhgef11	2.326401331	up	5.00E-05	Rho guanine nucleotide exchange factor (GEF) 11
	Cacna1s	2.784368194	up	0.01055	calcium channel, voltage-dependent, L type, alpha 1S subunit
	Mylk3	0.614572876	down	5.00E-05	myosin light chain kinase 3
Actin cytoskeleton regulation pathway	*Myh7b*	2.333782317	up	5.00E-05	myosin, heavy chain 7B, cardiac muscle, beta
	Git1	1.884015717	up	5.00E-05	G protein-coupled receptor kinase-interactor 1
	Myl9	1.838284422	up	5.00E-05	myosin, light polypeptide 9, regulatory
	Vav2	1.674959254	up	0.0032	vav 2 oncogene
	Arhgap35	1.508919666	up	5.00E-05	Rho GTPase activating protein 35
	Scin	0.657967395	down	0.0011	scinderin
	Mylk3	0.614572876	down	5.00E-05	myosin light chain kinase 3
	Itgb6	0.582105276	down	5.00E-05	integrin beta 6
	Mylk4	0.500210068	down	5.00E-05	myosin light chain kinase family, member 4

Note: Genes in purple are enriched in multiple pathways.

Table S2: FPKM value of 21 candidates from RNA-seq analysis.

Gene Name	FPKM value						Fold change
	Control 1	Control 2	Control 3	Rhau cKO 1	Rhau cKO 2	Rhau cKO 3	
Agpat1	17.4125	17.1212	16.8106	38.7097	38.6477	43.3917	2.384505
Dennd1a	6.1368	6.0198	7.4979	9.6839	12.9182	10.8394	1.579465
Gadd45a	5.4379	7.4125	4.9318	33.9369	24.5997	29.2844	5.010482
Hexim1	7.9776	9.1611	8.2851	19.5451	16.9922	18.6628	2.110929
Hlf	7.3734	6.1194	8.5769	12.0103	13.386	13.7627	1.809649
Kctd9	20.6432	16.0818	20.3073	32.1025	33.7619	35.538	1.646734
Kdm3b	7.3852	7.478	7.2246	20.2736	21.4525	19.663	2.809224
Msi2	56.4912	57.4344	55.4902	125.143	117.106	110.945	2.846813
Mta3	8.769	10.3538	8.9936	27.3591	30.0615	28.0608	3.121648
Nkx2-5	50.6458	57.6552	57.1996	169.995	173.528	193.459	3.424409
Phf23	10.0784	8.5465	8.7803	13.8256	14.8234	16.4545	1.515956
Psme3	24.1195	22.5487	26.8547	53.8604	56.2095	55.6544	2.287836
Rfx2	1.5703	1.6577	1.5963	3.0339	4.0824	3.0511	2.13484
Sec63	14.7829	16.0872	13.871	26.2241	23.2267	24.8791	1.631832
Set	77.5599	67.6268	79.2165	109.551	114.004	132.876	1.628913
Smarca4	14.45	14.2377	14.8785	21.0629	24.2177	23.1468	1.613025
Terf2	7.4068	7.2737	7.2981	14.4518	15.6139	15.6506	1.964547
Trim45	1.0144	1.4366	0.9234	4.0318	2.7336	3.7011	3.223934
Wdr59	2.6918	2.8276	3.1843	5.6788	5.1414	5.6185	1.88151
Xpr1	8.932	8.9761	9.6978	15.3263	15.9776	16.914	1.687886
Yap1	23.2677	24.5977	25.0828	49.1348	49.7438	48.2225	2.010165

Table S3: Information of antibodies.

Antibodies	Producer	Catalog number
Rabbit polyclonal anti-RHAU	ProteinTech	13159-1-AP
Rabbit polyclonal anti-SEC63	ProteinTech	13978-1-AP
Rabbit polyclonal anti-MTA3	ProteinTech	14682-1-AP
Rabbit polyclonal anti-GAPDH	Bioworld Technology	AP0063
Mouse monoclonal anti-β-Actin	Bioworld Technology	BS6007M
Rabbit polyclonal anti-α-Tubulin	Bioworld Technology	BS1699
Rabbit polyclonal anti-GFP	Santa Cruz	SC-8334
Mouse monoclonal anti-α-Actinin	Sigma-Aldrich	A7811
Rabbit monoclonal anti-YAP1	Cell Signaling Technology	14074S
Rabbit polyclonal anti-GADD45a	Cell Signaling Technology	4632
Rabbit polyclonal anti- KDM3B	Cell Signaling Technology	2621S
Rabbit polyclonal anti-HEXIM1	Abcam	ab25388
Goat polyclonal anti-NKX2-5	Abcam	ab106923
Rabbit monoclonal anti-BRG1	Abcam	ab110641
Rabbit polyclonal anti-HLF	Abcam	ab91630
Rabbit polyclonal anti-Histone H3	Abcam	ab5176
Rat monoclonal anti-Ki67	Dakocytomation	M724901
Mouse monoclonal anti-FLAG	Sigma	F1804
Goat anti-mouse IgG(H+L), Alexa Fluor 488	Jackson ImmunoResearch	115-545-166
Goat anti-rabbit IgG(H+L), cy3	Jackson ImmunoResearch	111-165-144
Goat anti-rabbit IgG(H+L) -HRP	Bioworld Technology	BS13278
Goat anti-mouse IgG(H+L) -HRP	Abcam	ab6789

Table S4: Primers used for qRT-PCR.

Genes	Forward	Reverse
**Mouse *Rhau***	TATATAGAGATGCAGCGTTTC	CACCTGATGGTTATTGATTAG
**Mouse *Gapdh***	CCTTCCGTGTTCCTACCCC	GCCCAAGATGCCCTTCAGT
**Mouse *β-Actin***	AGTGTGACGTTGACATCCGT	TGCTAGGAGCCAGAGCAGTA
**Mouse *α-Tubulin***	ATCGTGCACATCCAGATTGGC	AGTAGCTCCCAGCGCAGTCAA
**Mouse *Yap1***	TGAGATCCCTGATGATGTACCAC	TGTTGTTGTCTGATCGTTGTGAT
**Mouse *Hexim1***	GAGCCACTCTTGACAGAACATC	TGCAAGGACGCTCTCGATTG
**Mouse *Nkx2-5***	CAGTCTTGGGAGCTCAAGACTAACC	CAGATCCCCAAGCTTACTAGCAACTAC
**Mouse *Mta3***	GCCAGTTGAGGCCGACTTGAC	TGTCTCATTCAGCAGGGCCAC
**Mouse *Sec63***	CCCACAACTGTACCCTTACCT	ACTGCCCTTGTCAGAAATGTCG
**Mouse *Brg1***	CAAAGACAAGCATATCCTAGCCA	CACGTAGTGTGTGTTAAGGACC
**Mouse *Kdm3b***	TCAATATCCACTGGGTCTGTCG	TGCCCCTTTGCACACTTCAG
**Mouse *Hlf***	TCTTCATTCCCGATGATTTGA	GATTGCGATCTGGTTCTCCTT
**Mouse *Gadd45a***	CCGAAAGGATGGACACGGTG	TTATCGGGGTCTACGTTGAGC
**Mouse *Myh6***	AGTGCTTCGTGCCTGATGA	AACTTGGGTGGGTTCTGCT
**Mouse *Myh7***	GTCCAAGTTCCGCAAGGT	GGAGCTGGGTAGCACAAGA
**Mouse *Nppa***	GCTTCCAGGCCATATTGGAG	GGGGGCATGACCTCATCTT
**Mouse *Nppb***	CATGGATCTCCTGAAGGTGC	CCTTCAAGAGCTGTCTCTGG
**Rat *Rhau***	AAACGCAGAAGAACAAGG	CAGGAGCAAACCAGGATA
**Rat *Yap1***	GATGTACCATTGCCAGCAGGC	GGTCCTGCCATGTTGTTGTCTG
**Rat *Hexim1***	CGATGTCAGAGTCCCTCGTGC	GGCTTCCAATGTCGCTTCTTC
**Rat *Gapdh***	GGCACAGTCAAGGCTGAGAATG	ATGGTGGTGAAGACGCCAGTA
**Rat *β-Actin***	TTCCAGCCTTCCTTCCTG	GGTCTTTACGGATGTCAACG
**Luciferase**	GATTACCAGGGATTTCAGT	GACACCTTTAGGCAGACC

Table S5: Primers used for plasmid construction.

***Yap1* 5′-UTR EGFP**	F:ACCGGACTCAGATCTCGAGCTCGTTTGGGCGTCTGGAGCCAAAG;<br>R:GCCCGCGGTACCGTCGACTGCAGGCTGCGGCCTCGTTCGACGC
***Hexim1* 5′-UTR EGFP**	F:ACCGGACTCAGATCTCGAGCTCGAGGAAAAGAGGAGGAGGCG;<br>R:GCCCGCGGTACCGTCGACTGCAGGCTTAGTAGAGTTCTCTTCT
***Yap1* 5′-UTR EGFP mutation-1**	F: ACTCAGATCTCGAGCTCGTTTGGGCGTCTGGAGCCAA;<br>R:TGCTCGAGGCCGCCTCTTCTCTCCTCTTCTCTTCTCCGCGAGCTCCTTCC<br>TTC
**Mouse *Yap1* 5′-UTR EGFP mutation-2**	F:AGAAGAGGCGGCCTCGAGCAAGGAGTGCAGAGCGATGCGGGCGCGCG<br>TCG<br>CA; R: ACCATGGTGGCGACCGGTGGCTGCGGCCTCGTTCGACG
***Hexim1* 5′-UTR EGFP mutation-1**	F: ACTCAGATCTCGAGCTCGAGGAAAAGAGGAGGAGGCGGA;<br>R:AAGGGGTTAA<br>AGTCTCAGAGCTGCCACCTCTCACTTCCACGGGTGTCGCTCCA
***Hexim1* 5′-UTR EGFP mutation-2**	F:CTGAGACTTTAACCCCTTGTGAGCTCTGCGGCAGAAGATTTAACCCTTT<br>GTGG<br>ATTCGG; R: ACCATGGTGGCGACCGGTGGCTTAGTAGAGTTCTCTTCT
***Yap1* 5′-UTR Luci**	F: GGCCTAGGCTTTTGCAAAGTTTGGGCGTCTGGAGCCAAAG;<br>R: TGTTTTTGGCGTCTTCCATGGGGCTGCGGCCTCGTTCGACGC
***Yap1* Luci-CDS**	F: GAAAGATCGCCGTGTAATTGGAGCCCGCGCAACAGCCG;<br>R: AGCGGCCGGCCGCCCCGACCTATAACCACGTGAGAAAGC
***Yap1* Luci-3′-UTR**	F: GAAAGATCGCCGTGTAATAGCTGCAGGGAGCCACTCTG;<br>R: AGCGGCCGGCCGCCCCGACTTTAAGATTATAAAAATTGGT
***Hexim1* 5′-UTR Luci**	F: GGCCTAGGCTTTTGCAAAGAGGAAAAGAGGAGGAGGCG;<br>R: TGTTTTTGGCGTCTTCCATGGGGCTTAGTAGAGTTCTCTTC
***Hexim1* Luci-CDS**	F: GAAAGATCGCCGTGTAATATGGCCGAGCCACTCTTGAC;<br>R: AGCGGCCGGCCGCCCCGACCTAGTCGCCGAACTTGGAAAG
***Hexim1* Luci-3′-UTR**	F: GAAAGATCGCCGTGTAATACTGAAAACTTTTGGGGGGA;<br>R: AGCGGCCGGCCGCCCCGACTTTCAAAATAGACATTTAAT

Table S6: siRNA sequences

**Rat *Rhau***	Sense: GCACAUGGAUGAACGGCGAUU<br>Antisense: UCGCCGUUCAUCCAUGUGCUU
**Human *Rhau***	Sense: GGGAACUGCGAAGAAGGUAUU<br>Antisense: UACCUUCUUCGCAGUUCCCUU
**Negative control**	Sense: UUCUCCGAACGUGUCACGUTT<br>Antisense: ACGUGACACGUUCGGAGAATT

Table S7: Primers used for production of biotinylated RNA probe

***Yap1*-5′-UTR (or G4Sm-5’-UTR)**	F:CCAAGCTTCTAATACGACTCACTATAGGGAGAGTTTGGGCGTCTGGAGC;<br>R:GGCTGCGGCCTCGTT
***Yap1*-CDS**	F:CCAAGCTTCTAATACGACTCACTATAGGGAGAATGGAGCCCGCGCAACAG;<br>R:CTATAACCACGTGAGAAAGCTT
***Yap1*-3′-UTR**	F:CCAAGCTTCTAATACGACTCACTATAGGGAGAAGCTGCAGGGAGCCACTCT;<br>R:GAAATTCAAGAATAACCAGTTAAGT
***Hexim1*-5′-UTR (or G4Sm-5′-UTR)**	F:CCAAGCTTCTAATACGACTCACTATAGGGAGAGAGGAAAAGAGGAGGAGGC;<br>R:GGCTTAGTAGAGTTCTCTTCTGG
***Hexim1*-CDS**	F:CCAAGCTTCTAATACGACTCACTATAGGGAGAATGGCCGAGCCACTCTTG<br>R:CTAGTCGCCGAACTTGGAAAG
***Hexim1*-3′UTR**	F:CCAAGCTTCTAATACGACTCACTATAGGGAGAACTGAAAACTTTTGGGGGGA;<br>R:CAAAATAGACATTTAATGCAATC
P27-CDS	F:CCAAGCTTCTAATACGACTCACTATAGGGAGAATGTCAAACGTGAGAGTG<br>R: TTACGTCTGGCGTCGAAG
***Nkx2-5*-3′UTR**	F:CCAAGCTTCTAATACGACTCACTATAGGGAGAGGAAAGAGCCCGTTTGG<br>R: GCGTTAGCGCACTCACTT

Table S8: Abbreviations

RNA Helicase associated with AU-rich element	RHAU
RNA binding proteins	RBPs
G-quadruplex	G4
G4 resolvase 1	G4R1
AU-rich elements	AREs
embryonic day 7.5	E7.5
myocardial infarction	MI
the percentage of ejection fraction	EF%
the percentage of fractional shortening	FS%
left ventricular internal dimension at end-diastole	LVIDd
5 (7, 10, 14, 21) days after birth	P5 (7,10, 14, 21)
dilated cardiomyopathy	DCM
wheat germ agglutinin	WGA
gene ontology	GO
RNP immunoprecipitation	RIP
G4-forming sequences	G4S
coding sequence	CDS
positive control	PC
negative control	NC
Immunofluorescence	IF
fragments per kilobase of exon per million fragments mapped	FPKMs
Fold change	FC
standard deviation	SD
NK2 homeobox 5	NKX2-5
Yes1 associated transcriptional regulator	YAP1
hexamethylene bis-acetamide inducible 1	HEXIM1
natriuretic peptide type A	NPPA
natriuretic peptide type B	NPPB
myosin, heavy polypeptide 6, cardiac muscle, alpha	*Myh6*
myosin, heavy polypeptide 7, cardiac muscle, beta	*Myh7*
Phospho-histone H3	PH3
